# Thermo-Responsive Hyaluronan-Based Hydrogels Combined with Allogeneic Cytotherapeutics for the Treatment of Osteoarthritis

**DOI:** 10.3390/pharmaceutics15051528

**Published:** 2023-05-18

**Authors:** Alexandre Porcello, Paula Gonzalez-Fernandez, Annick Jeannerat, Cédric Peneveyre, Philippe Abdel-Sayed, Corinne Scaletta, Wassim Raffoul, Nathalie Hirt-Burri, Lee Ann Applegate, Eric Allémann, Alexis Laurent, Olivier Jordan

**Affiliations:** 1 School of Pharmaceutical Sciences, University of Geneva, CH-1206 Geneva, Switzerland; alexandre.porcello@unige.ch (A.P.); paula.gonzalezfernandez@unige.ch (P.G.-F.); eric.allemann@unige.ch (E.A.); 2 Institute of Pharmaceutical Sciences of Western Switzerland, University of Geneva, CH-1206 Geneva, Switzerland; 3 Preclinical Research Department, LAM Biotechnologies SA, CH-1066 Epalinges, Switzerland; annick.jeannerat@lambiotechnologies.com (A.J.); cedric.peneveyre@lambiotechnologies.com (C.P.); 4 Regenerative Therapy Unit, Lausanne University Hospital, University of Lausanne, CH-1066 Epalinges, Switzerland; philippe.abdel-sayed@chuv.ch (P.A.-S.); corinne.scaletta@chuv.ch (C.S.); nathalie.burri@chuv.ch (N.H.-B.); lee.laurent-applegate@chuv.ch (L.A.A.); 5 STI School of Engineering, Ecole Polytechnique Fédérale de Lausanne, CH-1015 Lausanne, Switzerland; 6 Lausanne Burn Center, Lausanne University Hospital, University of Lausanne, CH-1011 Lausanne, Switzerland; wassim.raffoul@chuv.ch; 7 Plastic, Reconstructive, and Hand Surgery Service, Lausanne University Hospital, University of Lausanne, CH-1011 Lausanne, Switzerland; 8 Center for Applied Biotechnology and Molecular Medicine, University of Zurich, CH-8057 Zurich, Switzerland; 9 Oxford OSCAR Suzhou Center, Oxford University, Suzhou 215123, China

**Keywords:** cartilage, cell therapy, hyaluronic acid, hydrogels, osteoarthritis, preclinical safety, thermo-responsive, viscosupplementation

## Abstract

Thermo-responsive hyaluronan-based hydrogels and FE002 human primary chondroprogenitor cell sources have both been previously proposed as modern therapeutic options for the management of osteoarthritis (OA). For the translational development of a potential orthopedic combination product based on both technologies, respective technical aspects required further optimization phases (e.g., hydrogel synthesis upscaling and sterilization, FE002 cytotherapeutic material stabilization). The first aim of the present study was to perform multi-step in vitro characterization of several combination product formulas throughout the established and the optimized manufacturing workflows, with a strong focus set on critical functional parameters. The second aim of the present study was to assess the applicability and the efficacy of the considered combination product prototypes in a rodent model of knee OA. Specific characterization results (i.e., spectral analysis, rheology, tribology, injectability, degradation assays, in vitro biocompatibility) of hyaluronan-based hydrogels modified with sulfo-dibenzocyclooctyne-PEG4-amine linkers and poly(N-isopropylacrylamide) (HA-L-PNIPAM) containing lyophilized FE002 human chondroprogenitors confirmed the suitability of the considered combination product components. Specifically, significantly enhanced resistance toward oxidative and enzymatic degradation was shown in vitro for the studied injectable combination product prototypes. Furthermore, extensive multi-parametric (i.e., tomography, histology, scoring) in vivo investigation of the effects of FE002 cell-laden HA-L-PNIPAM hydrogels in a rodent model revealed no general or local iatrogenic adverse effects, whereas it did reveal some beneficial trends against the development of knee OA. Overall, the present study addressed key aspects of the preclinical development process for novel biologically-based orthopedic combination products and shall serve as a robust methodological basis for further translational investigation and clinical work.

## 1. Introduction

Osteoarthritis (OA) is commonly characterized by the progressive structural and functional degeneration of articular cartilage tissue, with hypertrophic bone modifications and osteophyte formation, subchondral bone remodeling, and chronic synovial membrane inflammation [1,2]. For instance, knee OA is known to affect the entire joint via a multifactorial pathophysiological cascade [3,4]. Importantly, healthy cartilage tissue enables bone gliding, while simultaneously acting as an effective shock absorber [5]. Such intrinsic functional attributes of cartilage are normally enhanced by the local presence of synovial fluid (SF), naturally composed of lubricin and hyaluronic acid (HA), which provides additional cushioning and lubrication capacities to the load-bearing joints [3,6,7]. It is well established that qualitative pejoration of both SF composition/content and superficial chondral structures constitutes a key mechanism of knee OA progression [3,6,7,8]. Clinically, the affected patients develop localized joint pain, stiffness, motion limitation, and inflammation [3,9].

In classical pharmacotherapeutic approaches for OA-linked joint pain management and in addition to oral supplementation approaches, non-responders to oral antiphlogistics and analgesics are often prescribed with intra-articular viscosupplementation and/or corticoid infiltrations [10,11,12]. Therefore, exogenous HA-based hydrogels (i.e., polymeric network dissolved/dispersed in an aqueous solvent) have been extensively investigated as optimal synthetic SF substitutes designed for pain reduction and enhanced mobility recovery. Based on clinical reports of progressive physiological HA functional degradation and molecular weight (MW) distribution shifts in ageing patients, many therapeutic HA-based injectables for mild to moderate knee OA were developed using high-MW linear polymers of various origins [13,14]. However, the available aggregated clinical evidence suggested an absence of significant efficacy of classical HA-based viscosupplementation products in OA [15,16,17,18]. Such conclusions may possibly be methodologically linked to the analysis of inhomogeneous clinical studies and mechanistically linked to the rapid biopolymer clearance (i.e., oxidative, enzymatic, mechanical) from the treated joint following local administration. Therefore, the MW of hyaluronan-containing therapeutic agents is known to directly impact their functional attributes and their lymphatic drainage clearance rate, which can vary from days in the case of HA to 1–4 h for corticoids [17,18,19,20,21,22,23].

Notwithstanding the conflicting published reports on the clinical efficacy of HA-based knee viscosupplementation interventions in OA patients, the corresponding injectable products remain in standard rheumatology practice and are constantly being redeveloped by experienced industrial manufacturers. Therefore, hydrogel product efficacy optimization may rely on various HA chemical modifications (e.g., polymer crosslinking, chemical functionalization) or on the incorporation of appropriate excipients (e.g., carbohydrates, antioxidants) designed for physico-chemical attribute (e.g., product stability) enhancement or intra-joint residence-time improvement [10,14,20,24,25,26,27,28]. Similarly, the use of smart polymers has been extensively reported for potentially optimized hydrogel-based OA management, with leveraging of both technical and functional attributes of such products. Smart polymers, also known as stimuli-responsive polymers, are a class of materials that are able to respond to external stimuli such as local changes in temperature, pH, light exposure, and other environmental factors [29,30,31]. Thermo-responsive polymers exhibit a phase transition temperature in correspondence to which a drastic change in polymer solubility occurs [31]. In particular, water-soluble thermo-responsive polymers may transition from hydrophilic to hydrophobic as the system is heated above the ad hoc transition temperature (i.e., the lower critical solution temperature, LCST) [31]. Among such polymers, PNIPAM has been extensively exploited in diverse biomedical applications [14,25,26]. This is partly due to an LCST value of 32 °C, close to the human body temperature, and a low sensitivity of the phase transition to the local environmental conditions [14,27,31]. Above the LCST, PNIPAM polymers desolvate and condense, forming microgel domains. The HA-L-PNIPAM copolymer investigated in the present study undergoes a PNIPAM-driven physical crosslinking above the LCST, thanks to inter-chain interactions [14]. This in turn leads to an extended local residence time and to reduced enzymatic degradation of the implanted product. This property may improve the therapeutic effects of the treatment in the joint (i.e., prolongation of the exertion of the mechanical/physical effect of the product) [14,27].

Injectable combination products may also be considered for potentially enhanced OA management, with the incorporation of an appropriate active pharmaceutical ingredient (API, e.g., Cingal, HA-triamcinolone hexacetonide, Anika Therapeutics) or a biological agent (e.g., cell therapies, cellular derivatives), in order to exert an additional pharmacological or complex effect [10,28,32]. Therein, multiple biological material sources (e.g., autologous platelet derivatives, allogeneic stem and progenitor cells, cell secretomes, exosomes) have been proposed or clinically applied for knee OA, yielding some encouraging results (e.g., OA symptom alleviation as measured by WOMAC pain scores) [33,34,35,36,37,38,39,40,41]. Specifically, significant positive evolution of arthralgia, cellular and humoral immune responses, and patient histological parameters were reported for combinations of autologous platelets and HA, for example [33]. Currently, specific available insights into the pathophysiology of knee OA prompt the consideration and the further study of such injectable HA-based and biologically-based combination products for tentative enhanced management of the disease at its various stages [28,32,39]. Indeed, an optimal orthopedic therapeutic product could possibly comprise simple yet robust physical attributes (e.g., highly viscous gel in situ, adapted for local injection administration) supplemented with complex biological components (e.g., therapeutic cells or cell derivatives) providing ancillary or complementary effects [14,29,35]. Biologically-derived complex hydrogel systems thus bare the theoretical potential to simultaneously engage the complex causes and the multifactorial symptoms of progressive knee joint OA. While diverse approaches have been proposed to date, several aspects such as prototype scalability in industrial processing and clinical efficacy optimization remain at an advanced development stage [14,34].

Specifically, PNIPAM-containing thermo-responsive HA-based hydrogels and clinical grade FE002 primary allogeneic chondroprogenitor cell sources have both been previously proposed as tangible therapeutic options for the management of joint OA and of other chondral/osteochondral affections [14,27,42]. Intrinsic antioxidant attributes of alternative sources of lyophilized progenitor cells (e.g., FE002 progenitor tenocytes) have been reported and were identified as functionally relevant in the formulation of HA-based injectable products for tendinopathies (i.e., system functionalization, product stability enhancement) in particular [24,43]. Thus, for the further development of a potential injectable orthopedic combination product (i.e., indicated for mild to moderate knee OA management) incorporating both technologies (i.e., HA-L-PNIPAM copolymers and stabilized FE002 chondroprogenitors), several technical aspects (e.g., hydrogel synthesis upscaling and sterilization, FE002 cytotherapeutic material stabilization) were studied. Therefore, the aims of the present study comprised multi-step in vitro characterization experiments for several hydrogel combination formulas, with a strong focus set on critical functional parameters, as well as an in vivo assessment of their applicability, safety, and efficacy in an ACLT-hMnx rodent model of knee OA. Overall, the goal of the presented work consisted in the methodical experimental investigation of novel complex orthopedic combination products in advanced preclinical development, outlining important manufacturing and translational opportunities.

## 2. Materials and Methods

### 2.1. Reagents and Consumables Used in the Study

For the needs of the presented study, N-(3-dimethylaminopropyl)-N′-ethylcarbodiimide (EDC), N-hydroxysuccinimide (NHS), azide-terminated poly(N-isopropylacrylamide) (PNIPAM-N_3_; 15 kDa MW), hyaluronidase from bovine testes (Type VI-S), hydrogen peroxide 30% *w*/*w*, hydrochloric acid, sodium hydroxide, sodium chloride, Trypan blue exclusion dye, Trolox equivalent antioxidant capacity (TEAC) assay kits, ferric reducing antioxidant power (FRAP) assay kits, and all liquid media for cell culture were purchased from Sigma-Aldrich, St. Louis, MO, USA. WST-1 assay kits were purchased from Abcam, Cambridge, England. Laboratory-grade HA sodium salt (2200–2400 kDa MW) was purchased from Contipro, Dolní Dobrouč, Czech Republic. Sulfo-dibenzocyclooctyne-PEG4-amine (Sulfo-DBCO-PEG4-NH_2_) was purchased from Broadpharm, San Diego, CA, USA. Dialysis membranes (Biotech CE Dialysis Tubing, 300 kDa cutoff) were purchased from Repligen, Waltham, MA, USA. Pharmaceutical-grade purified water and phosphate-buffered saline (PBS) sterile buffer solutions were purchased from Laboratorium Dr. G. Bichsel, Unterseen, Switzerland. European pharmacopoeia (Ph.Eur.) grade saccharose was purchased from PanReac AppliChem, Darmstadt, Germany. Ph.Eur. grade dextran 40,000 was purchased from Pharmacosmos, Wiesbaden, Germany. Millipore Stericup and Millex GS filter-sterilizing membranes with 0.22 µm pores were purchased from Merck, Darmstadt, Germany. Nested 2R tubular clear glass vials for lyophilization were purchased from Schott, Mainz, Germany. Laminated elastomeric lyophilization stoppers were purchased from Flaver, Reinach, Switzerland. Lyophilization bags were purchased from Teclen, Oberpframmern, Germany. Flat-bottom 96-well microtitration plates and Eppendorf tubes were purchased from Greiner, Frickenhausen, Germany. Luer-Lok syringes and 18G blunt fill needles were purchased from BD, Franklin Lakes, NJ, USA, and from Schott, Mainz, Germany. Needles for injection with 27G bores were provided by Needle Concept, Biarritz, France.

### 2.2. Equipment and Instruments Used in the Study

Samples were lyophilized in a LyoBeta Mini freeze-dryer, Telstar, Terrassa, Spain, or in an Alpha 1-4 LDplus freeze-dryer, Christ, Osterode am Harz, Germany. Cell-containing samples were processed on a Countess 3 automated cell counter, Thermo Fisher Scientific, Waltham, MA, USA. Sample pH was determined using a SevenCompact Cond meter S230, Mettler Toledo, Greifensee, Switzerland. Sample osmolality was determined using an OsmoTECH XT, Advanced Instruments, Norwood, MA, USA. Sample centrifugation was performed on a Rotina 420R centrifuge, Hettich, Tuttlingen, Germany, or on a Legend Micro 21R centrifuge, Thermo Fisher Scientific, Waltham, MA, USA. Colorimetric measurements were performed on a Synergy Mx microplate reader, Biotek, Winooski, VT, USA. Rheological measurements were performed on a HAAKE Mars Rheometer, Thermo Fisher Scientific, Waltham, MA, USA. Karl Fisher residual humidity measurements were performed on a Coulometric KF Titrator Compact C30SD, Mettler Toledo, Greifensee, Switzerland. Injectability measurements were performed on a Texture Analyzer TA.XT. Plus instrument, Tracomme, Schlieren, Switzerland. Sample analysis by ^1^H NMR spectroscopy was performed on a Bruker Avance Neo 600 MHz NMR spectrometer, Bruker, Billeria, MA, USA. Tribology measurements were performed on an MCR 302 rotational rheometer, Anton Paar, Graz, Austria. CT scans were acquired using a microtomograph Bruker Skyscan 1076 instrument, Bruker, Billeria, MA, USA. Histological sample digitalization was performed using a NanoZoomer S60 imager, Hamamatsu, Hamamatsu City, Japan.

### 2.3. Lyophilized FE002 Primary Chondroprogenitor Preparation and Characterization

Primary human chondroprogenitors (i.e., FE002 clinical grade progenitor cell source) were procured and produced in vitro under the Swiss progenitor cell transplantation program and were made available for the present study in dry cell-pellet form. They were stored at −80 °C until use, as described elsewhere for alternative FE002 progenitor cell sources [24,42,43]. Processing of all biological materials was compliant with the Ethics Committee Protocol #62/07 of the University Hospital of Lausanne, Lausanne, Switzerland. All of the starting cellular materials had been serially expanded in vitro and had been harvested from confluent monolayers at passage levels of 6 and 7. For the needs of the study, the cells were thawed and were resuspended at 10^7^ cell equivalents/mL in lyoprotective solution, composed of sucrose (8% *m*/*v*) and of dextran 40,000 (2% *m*/*v*) in buffered aqueous solvent. The resulting cell suspension was serially dispensed in sterile 2R clear glass lyophilization vials, with a final filling volume of 0.75 mL/vial. Control formulations were prepared using only the lyoprotective solution, and the vials contained no cell-derived biological constituents (i.e., placebo formulation). Following initial freezing of the samples at −20 °C, further sample cooling was performed at −45 °C for 2 h in the freeze-dryer chamber. A vacuum of 0.1 mbar was established, and the multi-step primary drying phase was automatically performed over 39 h using a temperature ramp mode from −45 °C to 25 °C. A secondary drying phase was then performed under a vacuum of <0.02 mbar over 9 h using a temperature ramp mode from 25 °C to 20 °C. The obtained lyophilizates were crimp-sealed, labelled, boxed, and stored at 4 °C until further use. Descriptive controls of the obtained lyophilizates were performed by two-operator visual assessment and comprised gradings of lyophilizate cake presence, batch uniformity, cake aspect, and cake behavior. Cellular devitalization was confirmed by Trypan blue staining. Quantitative controls of the lyophilizates were performed and comprised sample uniformity of mass, relative remaining humidity levels, cake reconstitution time, and reconstituted sample osmolality and pH.

### 2.4. HA-L-PNIPAM Polymer Synthesis and Characterization

The polymer HA-L-PNIPAM was synthetized using the protocol of Porcello et al. (2022) with slight modifications [14]. Briefly, the HA salt (2200–2400 kDa MW) was dispersed in distilled water at ambient temperature. The final HA concentration was 0.3% *w*/*v*, and the system was homogenized by magnetic stirring for 2 h. Then, EDC (10 eq. COO^−^) and NHS (3 eq. COO^−^) were added at respective intervals of 15 min and the pH was adjusted to a value of 5.5, using a solution of HCl 0.1 M. Then, sulfo-DBCO-PEG4-NH_2_ (0.4 eq. COO^−^) was added. The reaction was allowed to proceed for 12 h at ambient temperature under magnetic stirring. The reaction mixture (HA-sulfo-DBCO-PEG4) was then dialyzed for 48 h using 300 kDa dialysis membranes (i.e., 3 times against 5% *w*/*v* NaCl and 3 times against purified water to remove EDC, NHS, and free sulfo-DBCO-PEG4) before being transferred to a round-bottom glass flask. PNIPAM-N_3_ (1 eq. DBCO) was added, and the reaction was allowed to proceed for 6 h at ambient temperature under magnetic stirring. The pH was then adjusted to a value of 7.0, using a solution of NaOH 0.1 M. The final product of synthesis was then dialyzed again and was frozen at −80 °C. The product was then lyophilized under a vacuum of 0.15 mbar at a constant set temperature of −80 °C for 48 h and was finally stored at 4 °C until further use. Analytical controls were performed by ^1^H NMR spectroscopy in D_2_O solvent after hyaluronidase enzymatic digestion of the intermediate product (HA-sulfo-DBCO-PEG4, DS_1_) and after enzymatic digestion of the final product (DS_2_), for elucidation of the chemical structures and the degrees of substitution (DS) [14].

### 2.5. HA-L-PNIPAM and HA-Based Hydrogel Formulation with Lyophilized FE002 Primary Chondroprogenitors

Lyophilized HA-L-PNIPAM was resuspended at 3.7% *w*/*v* in an aqueous solvent composed of distilled water and PBS in equal proportions. Linear HA (2200–2400 kDa MW) was resuspended separately at 1.0% *w*/*v* in the same aqueous solvent. Both hydrogel formulations were simultaneously autoclaved at 121 °C for 12 min before further use, in order to obtain sterile formulations. All characterization experiments were performed using steam-sterilized hydrogels. Then, the preparation of the combination products was realized by direct incorporation of the FE002 lyophilized chondroprogenitors or of the corresponding control lyophilizates following a method described previously [24]. Briefly, both hydrogel types were loaded into 3 mL Luer-Lok syringes mounted with 18G blunt-fill needles. Following dispensing of the hydrogels in the appropriate lyophilizate vials and after gentle mechanical homogenization using the tips of the needles, the reconstituted combination products were loaded into the Luer-Lok syringes for further experimental use.

### 2.6. Determination of the Intrinsic Antioxidant Capacity of Lyophilized FE002 Primary Chondroprogenitors

The antioxidant capacity of the lyophilized FE002 chondroprogenitors was assessed using two colorimetric assays, as previously described [24,43]. The Trolox equivalent antioxidant capacity (TEAC) assay kit was used according to the specifications of the manufacturer. Briefly, the lyophilizates were reconstituted in 300 µL of purified water. Following a gentle mechanical homogenization step, the samples were centrifuged at 5400× *g* at ambient temperature for 5 min, and volumes of 20 µL of clear supernatant from the samples were transferred to a 96-well microtitration plate. Then, volumes of 100 µL of the TEAC kit reaction mix were added to the samples, and the plate was incubated at ambient temperature for 10 min. The absorbance values of the samples were then determined at a wavelength of 570 nm. The absorbance value was determined to be proportional to the TEAC value of the samples, following the linear domain of the Beer–Lambert law and based on an experimental Trolox equivalent standard curve.

The ferric reducing antioxidant power (FRAP) assay kit was used according to the specifications of the manufacturer. Briefly, the lyophilizates were reconstituted in 300 µL of purified water. Following a gentle mechanical homogenization step, the samples were centrifuged at 5400× *g* at ambient temperature for 5 min, and volumes of 10 µL of clear supernatant from the samples were transferred to a 96-well microtitration plate. Then, volumes of 190 µL of the FRAP kit reaction mix were added to the samples, and the plate was incubated at 37 °C for 1 h in the dark. The absorbance values of the samples were then determined at a wavelength of 594 nm. The absorbance value was determined to be proportional to the FRAP value of the samples, following the linear domain of the Beer–Lambert law and based on an experimental ferrous equivalent standard curve.

### 2.7. Rheological Characterization of the Combination Products

The rheological behaviors of the sterile hydrogels (i.e., HA and HA-L-PNIPAM) and of the reconstituted combination products (i.e., HA with cells and HA-L-PNIPAM with cells) were determined by oscillatory rheology using a C35 2°/Ti Peltier heated cone-plate system and a hydrogel sample volume of 450 µL. The storage modulus (G′) and the loss modulus (G”) were determined at both 22 °C and 37 °C, with a constant oscillatory frequency of 0.5 Hz over 4 min. Three experimental replicates were used for all of the assays. Appropriate control samples were included. Shear stress was set to 3 N/m^2^ in all of the characterization experiments, in order to remain within the linear viscoelastic region (LVE). A sample hood was used during the measurements to minimize sample evaporation.

### 2.8. Combination Product Accelerated Degradation Assays with Rheological Readouts

Accelerated degradation assays were performed in vitro on the sterile hydrogels and on the reconstituted combination products under controlled oxidative stress or in the presence of hyaluronidase. The oxidative stress degradation assay was performed using 400 µL of hydrogel or combination product sample combined with 100 µL of hydrogen peroxide (H_2_O_2_ 30% *w*/*w*) for 1 h under incubation at 37 °C. The enzymatic degradation assay was performed using 400 µL of combination product sample combined with 100 µL of hyaluronidase 100 U/mL for 1 h under incubation at 37 °C. Endpoint analysis and data acquisition in oscillatory rheology were performed as described hereabove for basic rheological characterization of the samples. Control samples were systematically prepared with 100 µL of PBS instead of the hydrogen peroxide challenge item or the hyaluronidase challenge item.

### 2.9. Combination Product Rotational Tribology Characterization

The lubrication ability of the sterile hydrogels and of the combination product samples was studied in rotational tribology using a T-PTD 200 ball-on-three-pins tribology cell. The instrument was equipped with a SoLi glass ball of 12.7 mm in diameter and a sample holder with three cylindrically shaped polydimethylsiloxane (PDMS) pins of 6 mm in diameter, simulating the behavior of native cartilage surfaces. The measurements were performed at 37 °C on 700 µL of hydrogel sample with a normal force of 3 N, resulting in a maximum contact pressure of 290 kPa. Stribeck tests were conducted with a logarithmic sliding velocity increase (i.e., from 0.01 mm/s to 100 mm/s), while the normal force and the temperature were maintained as constants. Before each measurement, the samples were equilibrated in the instrument at 37 °C for 3 min and under 3 N of force. New PDMS pins were used for each measurement. The friction factor/coefficient μ, representing the interaction between both sliding surfaces, was measured as a function of the sliding velocity both during the increase and during the decrease in velocity.

### 2.10. Combination Product Injectability Assessment

The force injection profiles of the combination product samples were determined using syringes and needles appropriate for clinical viscosupplementation product administration in knee OA. Homogeneous reconstituted product sample volumes of 1 mL were loaded at ambient temperature in the syringes and were automatically extruded into air by a Texture Analyzer instrument set at a constant piston speed of 0.5 mm × s^−1^. The maximal applied pressure was set at 100 N. The force injection profile was recorded over the whole piston travel distance and was used to determine mean plateau injection force values.

### 2.11. Combination Product In Vitro Cytocompatibility with Primary Fibroblast-like Synoviocytes

The procurement, isolation, and in vitro culture of human fibroblast-like synoviocytes (HFLS) were performed as previously described, with slight adaptations [14]. Briefly, hip synovial membranes were collected from three adult male patients diagnosed with clinical OA, at the time of hip replacement surgery. Processing of all biological materials and information materials was compliant with the Ethics Committee Protocol #2017-02234 of the CCER of Geneva, Switzerland. Briefly, the harvested tissue samples were finely minced and were digested for 3 h at 37 °C under 5% CO_2_ incubation in a conserved 3 mg/mL collagenase IX-RPMI 1640 solution. After centrifugation at 200× *g* and supernatant removal, the resuspended cell pellet was cultured in vitro in monolayers at 37 °C and under humidified 5% CO_2_ incubation. The cell proliferation medium contained an equal ratio of RPMI 1640 and M199, with penicillin/streptomycin 1 IU/mL, 2 mM L-glutamine, and 20% fetal bovine serum. Non-adherent cells were removed after 12 h post-seeding. After 3 in vitro passages, confluent HFLS were treated in a 96-well microtitration plate (i.e., 10^4^ cells/well) with diluted combination product samples for up to 5 days of incubation. Then, adherent cells were tested for viability/metabolic activity using the cell proliferation reagent WST-1 according to the specifications of the manufacturer. Both of the native hydrogels (i.e., HA, HA-L-PNIPAM) and the corresponding combination products (i.e., HA + Cells, HA-L-PNIPAM + Cells) were tested in the WST-1 setup. Cell culture medium was used as a positive control, and hypotonic aqueous solvent (i.e., mimicking the hydrogel reconstitution solvent) was used as an internal control.

### 2.12. Combination Product In Vivo Efficacy Assessment in a Rodent Model of Knee Osteoarthritis

#### 2.12.1. Knee Osteoarthritis Model and Surgical Procedure

The described in vivo work and the related analyses were performed in collaboration with Atlantic Bone Screen, St-Herblain, France. The animal model used for this study was in line with the European Directive 2010/63/UE of 2010 and was authorized by the local Ethical Committee and the French Ministry for Education and Research under the agreement number APAFIS#20051. Forty-three animals were included in the study and were randomly allocated to one of five groups. Knee OA was induced in ten-weeks-old male Sprague Dawley rats by performing an anterior cruciate ligament transection (ACLT) and hemi-meniscectomy (hMnx) surgical procedure on the right posterior limbs of the animals. Pre-emptive analgesia with buprenorphine supplemented with meloxicam was subcutaneously injected 30 min before the surgery. The animals were fully anesthetized by a mix of air and isoflurane (i.e., air 0.5 L/min; isoflurane 5%), maintained under narcosis via an air flow of 0.5 L/min and 2–3% isoflurane, and were placed on a warm plate during the surgery procedure. The right knee of each animal was prepared for surgery and was exposed via a para-patellar approach. A skin section was performed laterally to the patella. Some lidocaine solution was applied locally on the articular capsules immediately prior to the incision. The anterior cruciate ligament (ACL) was sectioned, and the anterior part of the medial meniscus was resected before repositioning of the patella. Both the capsule and the skin were sutured, and further analgesic injections were administered around 6 h after the surgery. Analgesic injections were administered over the course of the following days according to the observed clinical state of the operated animals. Thorough post-operative follow-up was carried out to ensure uncomplicated wound healing, sufficient analgesia, and general well-being of the animals. Thereafter, the general clinical state of the animals was monitored daily.

#### 2.12.2. Investigative Product Groups and Product Administration Modalities

The study animals within each group (i.e., 8 or 9 animals per group) each received a single dose of investigative product, which was composed of PBS (i.e., operated control group), a sterile hydrogel composed of linear HA with or without lyophilized FE002 chondroprogenitors, or a sterile hydrogel composed of HA-L-PNIPAM with or without lyophilized FE002 chondroprogenitors. The investigative products were administered under general animal anesthesia by intra-articular injection into the knee joint, nine weeks post-surgery after the ACLT-hMnx procedure. Syringes mounted with 30G needles were used to inject a single dose of 50 µL of investigative product into the articular cavity. One syringe was used per animal. Thereafter, the general clinical state of the animals was monitored daily.

#### 2.12.3. Terminal Procedure and Sampling of Posterior Knee Joints

Following a period of 91 days post-ACLT-hMnx surgery, the animals were euthanized following the study protocol by cervical dislocation under general anesthesia. Local necropsy evaluation was performed by exposing the knee joint. Macroscopic evaluations were performed and recorded, focusing on local tissue reaction and on the presence of the investigative products and/or degradation residues. Operated knee joints (i.e., right knees) and non-operated knee joints (i.e., contra-lateral left knees) were further harvested. The samples were immediately stored in a 10% formalin solution for a minimum of 72 h and a maximum of two weeks, to avoid over-fixation and tissue damage.

#### 2.12.4. Micro-CT Data Acquisition and Data Analysis by Scoring

During the sample fixation step in formalin, the harvested knees were scanned by micro-CT. The following parameters were used: 70 kV for voltage, 145 mA for tension, a resolution of 9 µm, a rotation angle of 0.6°, an averaging of 1, with a filter of 0.5 mm in diameter, and a bed of 35 mm. Reconstruction, realignment, and selection of longitudinal sections at the center of the knees, as well as at the transverse sections in the tibial plateau and the femoral condyle, were performed to score the subchondral bone lesions. On CT-scan images, the severity of OA was blindly evaluated and scored, with scores ranging from none (score 0) to extended (score 2) for osteophyte presence, from none (score 0) to more than half of the subchondral or cortical epiphyseal bone (score 3) for bone sclerosis, and from none (score 0) to more than half of the area (score 4) for bone cysts.

#### 2.12.5. Histological Analyses and Histopathological Chondral Defect Grading

Following the micro-CT analyses, all of the collected samples were decalcified in an EDTA-based solution before trimming, dehydration, and paraffin embedding. Samples were prepared and oriented to be cut in the coronal plane to enable the exhibition of the medial and lateral faces of the femoral condyle and of the tibial plateau. Two slides were produced for each sample, one being then stained with Hematoxylin/Eosin/Saffron (HES) and one being stained with Toluidine Blue (TB). Digitalized slides were obtained with a NanoZoomer S60 imager, using the 20× magnification setting. For each sample, the digitalized slides were blindly evaluated and scored by two experienced veterinarian histopathologists. The cartilage structure and the proteoglycan content parameters were graded using the Osteoarthritis Research Society International (OARSI) grading scheme described for guinea pigs, according to previous publications [44,45].

#### 2.12.6. Histomorphometry Evaluation of Osteoarthritis Progression

Quantitative and semi-quantitative histomorphometric evaluation of knee OA was performed on the medial tibial plateau of the considered joints. Methods and scoring were both based on the OARSI recommendations [44]. Total cartilage degeneration width was defined as the measurement of the width of articular cartilage affected by any type of degenerative change and expressed as a percentage relative to the total width of the considered articular surface. Significant degeneration width was defined as the measurement of the width of the articular surface in which 50% or more of the thickness (i.e., from surface to tidemark) was seriously compromised and expressed as a percentage relative to the total width of the considered articular surface. The articular surface of the medial tibial plateau was divided into three zones (i.e., Z1, Z2, and Z3) to evaluate cartilage loss and loss of staining in different load-bearing areas. Cartilage degeneration in each zone was scored as ‘none’ to ‘severe’ (i.e., numerical values of 0 to 5), as described hereafter. No degeneration was graded as 0, minimal degeneration around 5–10% of the total projected cartilage area affected by matrix or chondrocyte loss was graded as 1, mild degeneration (around 11–25%) was graded as 2, moderate degeneration (around 26–50%) was graded as 3, marked degeneration (around 51–75%) was graded as 4, and severe degeneration (greater than 75%) was graded as 5. A 3-zone sum for cartilage degeneration was also calculated by summing the values obtained for each zone. The maximum 3-zone sum for the medial tibia was 15. Cartilage matrix loss width was expressed in µm, considering the width of cartilage matrix loss along the projected cartilage surface (i.e., 0% depth), at the tidemark (i.e., 100% depth), and at the midpoint of the cartilage thickness (i.e., between the surface and the tidemark, at around 50% depth).

### 2.13. Statistical Analyses and Presentation of Experimental Data

For the statistical comparison of average values from two datasets, an unpaired Student’s T-test was applied, after assessing whether the data followed a normal distribution. A *p*-value < 0.05 was retained as a base for statistical significance determination. For the statistical comparison of values from multiple quantitative datasets from experiments where multiple variables applied, a one-way ANOVA test was performed, and it was followed (when appropriate) by a post hoc Tukey’s multiple comparison test or was substituted by a Kruskal–Wallis one-way analysis of variance (i.e., for the analysis of non-parametric variables/data such as gradings). The statistical calculations and/or data presentation were performed using Microsoft Excel (Microsoft Corporation, Redmond, WA, USA), Microsoft PowerPoint, and GraphPad Prism v. 8.0.2 (GraphPad Software, San Diego, CA, USA).

## 3. Results and Discussion

### 3.1. Lyophilized FE002 Chondroprogenitors Possess Intrinsic Antioxidant Properties and Functionalize Hydrogels through Stability Enhancement

In contrast to classical cytotherapeutic approaches for chondral lesions (i.e., use of viable and chondrogenic cells), the biological materials described herein were processed into a stable and devitalized form (i.e., cell lyophilizates) [46]. This approach was pursued based on previous reports and publications on alternative FE002 primary progenitor cell stabilization, where various functional parameters (e.g., intrinsic antioxidant activity) were shown to be conserved in the lyophilizates, as compared to freshly harvested cells [24,47]. Importantly, this approach was deemed foundational for the tangible further development of widely available cell-based or cell-derived therapies [43]. Indeed, while the efficacy of freshly cultured and viable chondrocytes has been clinically investigated for several decades, high manufacturing and logistical constraints drastically limit the number of potential benefitting patients [48,49]. Therefore, in addition to the allogeneic nature of the described lyophilized FE002 chondroprogenitors (i.e., low immunogenicity, possibility of standardizing the manufacturing workflows), the conferred biological material temperature stability opens diverse logistical and final formulation opportunities (e.g., off-the-self therapeutic products) [24].

As concerns the retained lyophilization protocol, the described method and cycle were established during previous in-house studies on the optimal stabilization of therapeutic cellular components, with optimal preservation of function as a critical objective (i.e., maximization of biological sample quality attributes) [24,43,47]. Therein, various cryoprotective and lyoprotective excipients (e.g., sugars) were tested and various lyophilization cycle parameters were investigated [43,47]. Overall, the described lyophilization recipe was established as an optimal approach for the temperature stabilization of functional cell-based and cell-derived materials in general [43]. Therefore, no further or specific lyophilization process/cycle optimization studies were carried out in the present work, as the recipe had been previously and repeatedly validated. As part of future work on the scalability and transposition of the cell lyophilization phase in particular, process and recipe simplifications may potentially be implemented (i.e., regulation of time and energy resources), but only after being duly validated against historically proven data and processes.

**Figure 1:**
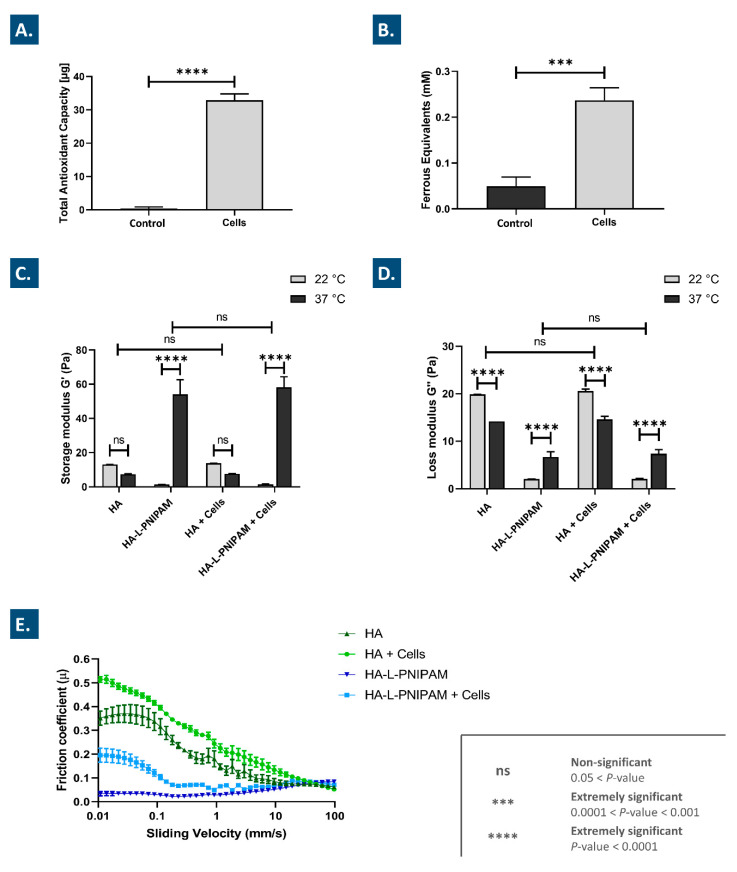
Antioxidant properties assessed as the TEAC (**A**) and the FRAP (**B**) of the lyophilized FE002 chondroprogenitor preparations. Results are expressed as means with standard deviations as error bars for six replicates. Storage modulus G′ (**C**) and loss modulus G″ (**D**) values of hydrogel combination products at 22 °C and 37 °C, determined by oscillatory rheology at a frequency of 0.5 Hz. Results are expressed as means with standard deviations as error bars for six replicates. Stribeck curves of the hydrogels and of the combination products at 37 °C and with a sliding velocity increase from 0.01 mm/s to 100 mm/s (**E**). Results are expressed as means with standard deviations as error bars for four replicates. Extremely significant statistical differences (i.e., **** or *p*-value < 0.0001; *** or 0.0001 < *p*-value < 0.001) or no statistical differences (ns) were found between the presented mean values. FRAP, ferric reducing antioxidant power; HA, hyaluronic acid; ns, non-significant; TEAC, Trolox equivalent antioxidant capacity.

Following experimental sample lyophilization, two-operator gradings of the obtained vial batches enabled confirmation of structurally coherent cake formation (Table S1). Then, the assessment of the intrinsic antioxidant properties of the stabilized FE002 chondroprogenitor preparations revealed significant TEAC and FRAP values of the cell-laden samples, as compared to the control samples (Figure 1A,B). These results are in line with previous reports on the intrinsic antioxidant capacity (i.e., critical functional attribute) of lyophilized FE002 progenitor tenocytes prepared using a similar manufacturing workflow [24,43].

In contrast to viable therapeutic chondrocytes, for which critical functionality is classically defined as the remaining or the reacquired chondrogenic potential in the final product formulation, the antioxidant capacity of the lyophilized FE002 chondroprogenitors is found to play important functional roles (e.g., system stabilization and internal protection) within HA-based hydrogels [24,49]. Specifically, it was shown that the TEAC values of the considered FE002 chondroprogenitor lyophilizates were comparable in terms of absolute values with the data previously reported on lyophilized FE002 progenitor tenocytes (Figure 1A) [24]. It is noteworthy that the FRAP and TEAC assays could not be performed with HA or HA-L-PNIPAM-containing samples (i.e., characterization of the lyophilizates instead), due to the viscosity of the reconstituted formulations. Overall, the presented experimental data confirmed that the considered FE002 chondroprogenitors could be obtained in a stable (i.e., lyophilized) and functional (i.e., antioxidant) form, which is adapted for final formulation in HA-based injectable hydrogels.

### 3.2. Thermo-Responsive Hydrogels May Be Simply Synthetized and Autoclave-Sterilized before Inclusion of Cell Lyophilizates

The main technical and functional advantages of working with thermo-responsive hydrogels for OA reside in an easy injection through standard orthopedic needles, coupled with significantly increased in situ viscosity of the hydrogel inside the joint [14]. In order to prepare HA-based thermo-responsive hydrogels with improved potential for industrial manufacturing transposition, scaling up (i.e., polymer synthesis, hydrogel terminal sterilization), and preclinical translation, modifications were introduced to methods previously reported by Porcello et al. [14,27].

The chemistry synthetic routes for the considered HA-L-PNIPAM copolymers are presented in Figure S1. Based on previous in-house work, sulfo-DBCO-PEG4-NH_2_ was linked to the HA backbone via an amidation reaction (Figure S1A). Then, PNIPAM-N_3_ was linked with the DBCO group via copper-free azide–DBCO click chemistry (Figure S1B). The final polymeric product was dialyzed against 5% *w*/*v* NaCl in distilled water using a dialysis membrane with a 300 kDa mass cutoff and was lyophilized, in order to easily disperse the copolymers and to obtain the desired concentration. Then, once the sterile hydrogels were available (i.e., after autoclaving), the various combination products were prepared by direct incorporation of the cell lyophilizates. A summary of the composition of the various considered hydrogels and combination products is presented in Table 1.

Analytical control results obtained by ^1^H NMR spectroscopy are presented in Figure S2. The degree of substitution resulting from the amidation reaction (i.e., DS_1_) was calculated using the aromatic protons of DBCO, and the obtained DS_1_ value was 10.7%. The DS resulting from the copper-free azide–DBCO click chemistry (i.e., DS_2_) was obtained by integration of the CH_3_ groups of PNIPAM and of the aromatic protons of DBCO. The obtained DS_2_ value was 59.9%. The HA contents, as well as sulfo-DBCO-PEG4-NH_2_ and PNIPAM contents, in the final hydrogel products could be calculated from the obtained DS values in ^1^H NMR (Figure S2, Table 1). Furthermore, extensive HA-L-PNIPAM polymer characterization (i.e., ^1^H NMR, FT-IR, UV-Vis) was previously reported, and the results obtained in this study are in line with historically validated data [14]. Formulations containing HA-L-PNIPAM were prepared by dispersing the lyophilized co-polymer at a gross concentration of 3.7% *w*/*v*, corresponding to a net concentration of 1% *w*/*v* of HA. This final concentration was chosen to obtain identical net HA concentrations in all of the considered hydrogels (Table 1). It is noteworthy that hydrogel formulations containing linear HA and HA-L-PNIPAM were submitted (i.e., prior to the incorporation of the FE002 cell lyophilizates) to the same steam sterilization cycle using a temperature of 121 °C for 12 min. Measurable and significant degradation of the hydrogels (i.e., represented by sample viscoelastic properties) occurring during the sterilization step is presented in Figure S3.

Linear HA-based hydrogels were prepared in parallel to the HA-L-PNIPAM hydrogels as experimental controls (i.e., non-thermo-responsive systems, Table 1). Hydrogels loaded with lyophilizates containing no cells (e.g., only the sucrose/dextran mix) were included as controls, for the assessment of the effect of the FE002 cells on system parameters and functions. Experimentally, the HA-L-PNIPAM-based hydrogels presented technical advantages over the linear HA-based hydrogels during the extemporaneous lyophilizate reconstitution phase, due to lower system viscosity at ambient temperature (i.e., 20–25 °C), which led to facilitated manual sample homogenization. Nevertheless, all of the combination products were easily prepared in less than 5 min.

### 3.3. Combination Products Display Optimal Thermo-Responsive Behaviors and Lubricating Effects In Vitro

A critical consideration pertaining to component compatibility within the studied combination products consisted in the conservation of the thermo-responsive and lubricating behaviors of the system following the incorporation of the stabilized FE002 cells. Therefore, the viscoelastic properties of the native hydrogels and of the various combination products were determined rheologically at 22 °C and at 37 °C, using an appropriate oscillatory frequency of 0.5 Hz (i.e., simulating human walking conditions, Figure 1C,D). The results clearly outline a significant increase in both of the considered moduli (i.e., 40 × increase factor for G′ and 3 × increase factor for G″) for HA-L-PNIPAM-based formulations at 37 °C as compared to the groups analyzed at 22 °C (Figure 1C,D). At low temperatures (i.e., with respect to the LCST), PNIPAM orders itself in solution in order to form hydrogen bonds with the already arranged water molecules. At higher temperatures, the entropy term dominates, causing PNIPAM to release water and leading to phase separation. The exact mechanism leading to the recorded increase in G′ at high temperatures in the resulting hydrogel is probably mainly due to the conformation adopted by the nanostructured gel containing HA and PNIPAM, wherein microgel structures are entangled in the gel network, thereby protecting the hydroxyl groups of HA from degradation [14]. Importantly, no significant differences in the viscoelastic behaviors were observed between the cell-containing and the cell-free samples at either of the considered temperatures (Figure 1C,D). These results confirm the specific thermo-responsive behavior of the HA-L-PNIPAM-based hydrogel systems as independent from the presence of the lyophilized FE002 chondroprogenitor cells [14].

As regards the rheological behavior of the linear HA-based samples, decreases were observed in both moduli at 37 °C as compared to the values obtained at 22 °C, as expected for a non-crosslinked polymer solution. Specifically, the decrease was not found to be significant for G′ but was found to be significant (i.e., approximatively 25% decrease at 37 °C on average) for G″ (Figure 1C,D). In the HA-L-PNIPAM groups, no significant differences in viscoelastic behaviors were observed between the cell-containing and the cell-free samples (Figure 1C,D). Overall, the experimental viscoelastic behaviors were found to be significantly different between the HA-based and HA-L-PNIPAM-based formulations. Namely, G′ mean values at 37 °C were determined to be 7 times higher for the HA-L-PNIPAM groups, yet G″ mean values were determined to be 2 times lower than those of the HA groups (Figure 1C,D). These specific differences confirmed that the HA-based formulations acted as viscous fluids (i.e., G″ > G′), whereas the HA-L-PNIPAM-based formulations acted as viscoelastic gels (i.e., G′ > G″). Finally, it was shown that post-sterilization (Figure S3), both of the considered hydrogel types expressed viscoelastic properties/attributes (i.e., viscosity values at a given temperature) comparable in range to those of available commercial viscosupplementation products (i.e., Ostenil, TRB Chemedica, Geneva, Switzerland, or Monovisc, Anika Therapeutics, Bedford, MA, USA) [14,50,51]. Specifically, the net concentration of HA was constant in all of the considered hydrogel product formulas (Table 1). While the rheological profiles were found to be different between the thermo-responsive and the non-thermo-responsive hydrogel products, the absolute values (i.e., individually determined at defined temperatures) remained in comparable ranges (i.e., between 60 and 2 Pa for both moduli). Specifically, such absolute values were comparable with those of commercially available products (e.g., Ostenil, Euflexa, Synvisc), which are characterized by values between 95 Pa and 0.03 Pa for both moduli [52].

The lubrication capabilities (e.g., as assessed by the measurement of sample frictional behaviors in the Stribeck curves) of viscosupplementation products constitute a main functional attribute, along with the shock-absorbing capacity. Tribological characterization of the considered combination products indicated that up to a sliding velocity of 25 mm/s, linear HA-based FE002 cell-laden samples and HA-L-PNIPAM-based samples possessed the highest and the lowest coefficients of friction, respectively (Figure 1E). Furthermore, it was shown that with sliding velocities above 1 mm/s, the coefficients of friction of both HA-L-PNIPAM-based samples rose, while the curves of both HA-based samples dropped to relatively lower values (Figure 1E). The overall Stribeck curve characteristics of the HA-based FE002 cell-laden samples were found to be similar to those of the HA-based cell-free samples, albeit with higher coefficients of friction in the lower sliding velocity range, suggesting that both samples could behave similarly in a knee viscosupplementation application (Figure 1E). Similar conclusions were drawn for cell-laden and cell-free HA-L-PNIPAM-based samples and confirmed their applicability for viscosupplementation purposes in OA.

According to the literature on OA, injectable viscosupplementation product lubricating abilities are more predictive of the clinical outcomes than the viscoelastic properties of the same product [53]. Aggregation of the obtained tribological data demonstrated that HA-L-PNIPAM-based formulations presented specific Stribeck curve characteristics and were thus expected to behave differently in the joint as compared to linear HA-based formulations (Figure 1E). Specifically, local accumulation and adhesion of HA-based polymers at the articular surface is known to allow for viscous surface layer development and facilitation of viscous boundary lubrication [53]. Due to the microgel structure formation in the HA-L-PNIPAM-based formulations above the lower critical solution temperature (LCST), such mechanisms may procure additional advantages over linear HA-based formulations [14]. Overall, it may be concluded that thorough characterization of in vitro hydrogel properties and behaviors is mandatory for the effective optimization of novel HA-based derivatives, despite the numerous limitations on modelling and mimicking the in vivo situation in the joint.

### 3.4. Combination Products Present Significantly Improved Stability against Oxidative Degradation

**Figure 2:**
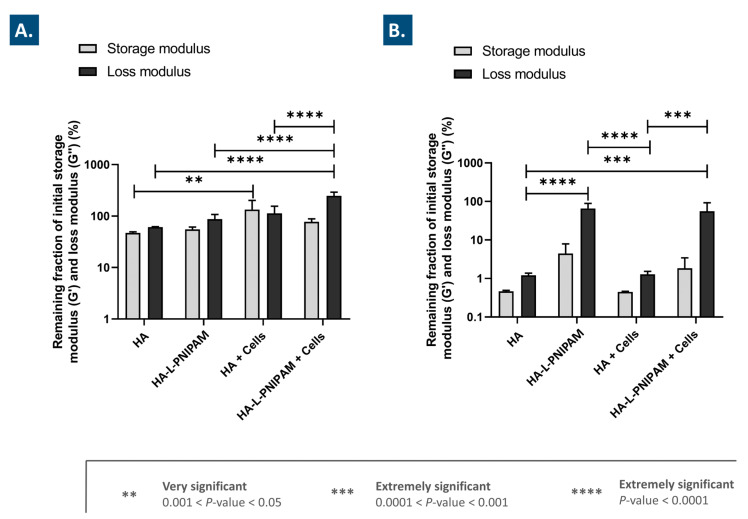
Storage modulus (G′) and loss modulus (G”) of the considered combination products, normalized to initial values and expressed as percentages, after one hour of exposure to H_2_O_2_ (**A**) or to hyaluronidase (**B**), determined by oscillatory rheology at a frequency of 0.5 Hz and at 37 °C. Results are expressed as means with standard deviations as error bars for three replicates. Extremely significant statistical differences (i.e., **** or *p*-value < 0.0001; *** or 0.0001 < *p*-value < 0.001), very significant statistical differences (i.e., ** or *p*-value 0.001 < *p* < 0.05), or no statistical differences (ns) were found between the presented mean values. HA, hyaluronic acid; PNIPAM, poly(N-isopropylacrylamide).

It is well established that in knee OA, endogenous or exogeneous HA is mainly degraded by hyaluronidases and by the action of local free radicals [19,20,24]. To assess the stability of the considered combination products against enzymatic and oxidative degradation, challenge assays coupled with rheological readouts were performed. After 1 h of exposure to a high concentration of H_2_O_2_ for the in vitro simulation of oxidative stress, the linear HA-based formulations presented a G′ decrease of 53% and a G″ decrease of 39% (Figure 2A).

However, the linear HA-based FE002 cell-laden formulations displayed a completely different behavior, with an increase in both of the considered moduli (i.e., 34% for G′ and 13% for G″, Figure 2A). These results suggest that the addition of the stabilized FE002 chondroprogenitors significantly protected the hydrogels against oxidative degradation, possibly partly via an intrinsic antioxidant effect exerted by the cells (Figure 1A,B and Figure 2A). Moreover, these results are congruent with previous reports on the rheological-behavior-modifying capabilities of lyophilized FE002 progenitor cells in oxidative challenge assays [24]. Specifically, it was previously hypothesized that a form of chemical crosslinking, mediated by oxidative stress, occurred between the polymer backbone and the proteinic constituents of the FE002 cellular lyophilizates, resulting not only in G′ stabilization but in rising G′ values [24]. The exact mechanism of interaction between both constituents under strong oxidative challenge is currently incompletely understood, and mechanistic research is ongoing [24,43].

In comparison, the HA-L-PNIPAM-based hydrogel samples submitted to the oxidative challenge presented a decrease in G′ of 45% and a decrease in G″ of 13% (Figure 2A). Generally, the remaining fractions of the initial moduli mean values were found to be slightly higher for the cell-free HA-L-PNIPAM-based samples than for the cell-free HA-based samples. However, the combination products containing HA-L-PNIPAM and FE002 cells presented a decrease of 23% in G′ but a significant increase in G″ of 147% (Figure 2A). Similarly to the case of the HA-based formulations, the combination of FE002 cells with HA-L-PNIPAM resulted in an improved system resistance to oxidative stress. Previous in vitro oxidative challenges had been reported for various alternative HA-L-PNIPAM-based formulations and had yielded similar results, yet the experimental H_2_O_2_ concentrations were only 50% of those used herein [14]. Mechanistically, the 2.5-fold increase in G″ for HA-L-PNIPAM-based cell-laden samples may potentially be simultaneously explained by the intrinsic antioxidant properties of the lyophilized FE002 cells; a favorable conformation adopted by the nanostructured gel network, which could protect the hydroxyl groups of the HA backbones; and an interaction of this network with the lyophilized FE002 cells [14,24]. Overall, these experimental results underline a clear combination product system-stability-enhancing function of the considered lyophilized FE002 chondroprogenitors.

### 3.5. The Use of HA-L-PNIPAM Efficiently Improves HA-Based Hydrogel Resistance toward Enzymatic Degradation

After in vitro exposure to hyaluronidases, the remaining fractions of the initial moduli of the HA-based cell-free samples were determined to be lower than 2% (Figure 2B). No significant differences in behavior were outlined for the HA-based FE002 cell-laden samples (Figure 2B). In comparison, the HA-L-PNIPAM-based cell-free samples displayed remaining fractions of the initial G′ modulus of 4% and remaining fractions of the initial G″ modulus of 66% (Figure 2B). However, the HA-L-PNIPAM-based FE002 cell-laden samples displayed remaining fractions of the initial G′ modulus of 2% and remaining fractions of the initial G″ modulus of 56% (Figure 2B). Therefore, the presence of the FE002 cells slightly quantitatively decreased the viscoelastic properties of the system, yet these differences were not found to be significant. Overall, it could be concluded that for both of the considered hydrogel types, the presence of the lyophilized FE002 chondroprogenitors did not significantly influence the system degradation induced by hyaluronidases. In contrast, the HA-L-PNIPAM-based products displayed significantly enhanced resistance to enzymatic degradation, as compared to the linear HA-based samples (Figure 2B). Two main explanations for the observed reduction in enzymatic degradation of the HA-L-PNIPAM-based systems are the reduction in the number of available HA carboxyl groups, which are known to be recognized by hyaluronidases, and the steric hindrance, which is established in the microgel structures of the system above the LCST [14,54]. Overall, it was concluded that in addition to the enhanced administration-related functional attributes of HA-L-PNIPAM-based hydrogels (i.e., as compared to linear HA), significant intrinsic protective effects could also be harnessed for maximization of product efficacy in vivo.

### 3.6. The Studied Combination Products Display No Cytotoxicity In Vitro and Are Easily Injectable

For the assessment of the considered combination products from a further translational point-of-view, both the cytocompatibility and the injectability of the various samples were studied. Firstly, in order to assess the cytocompatibility and the potential cytotoxicity of the considered combination products, a WST-1 in vitro cellular assay with primary HFLS indicated that all of the considered samples produced results that are not statistically different from the controls at the 5-day timepoint (Figure S4).

Secondly, the results of the in vitro injectability assays confirm that the various combination product samples could be easily injected through 23G needles. Specifically, it was outlined that the piston force required to inject the various preparations reached a constant plateau in the force injection profiles (Figure S5). Quantitative data confirmed that all of the necessary mean force levels required to inject the various hydrogel preparations were inferior to the 64 N limit, which is known to constitute the upper limit of easily injectable or syringeable hydrogel products (Figure S5) [55,56]. Additionally, HA-L-PNIPAM-based formulations could be injected with thinner needles during the in vivo study (i.e., 30G needles), indicating that additional margins of adaptability exist as regards clinical delivery modalities. Overall, it was found that all of the considered formulas behaved appropriately within the two retained translation-oriented assays, leading to the performance of the in vivo study in rodents.

### 3.7. The Studied Combination Products Did Not Cause Adverse Reactions In Vivo and Procured Multi-Parameter Beneficial Trends in a Rodent Model of Knee OA

A rat model of knee OA was selected for the in vivo study of the considered combination products, due to ease of animal handling and the technical possibility to induce partial chondral defects (i.e., presence of thicker cartilage than in mice) [57]. The ACLT-hMnx procedure was retained, as the ACLT surgery is known to produce the greatest effects as regards joint instability and the ACLT-hMnx combination enables the effective acceleration of OA progression in the animal model [45,58]. Nevertheless, the retained model was characterized by a relatively slow progression of knee OA, resulting in moderate structural defects as compared to the well-studied and surgically induced medial meniscal tear (MMT) model [44,59]. As reported, the animal study was performed at the facilities of Atlantic Bone Screen in St-Herblain, France.

The general synopsis of the animal study is presented in Figure S6. During the study, animal body weight and general clinical aspects (i.e., knee infection and oedema, articular joint inflammation, and global reaction to the combination products) were monitored twice per week. As expected, a general increase in animal body weight was observed over the course of the in vivo study (Figure S7). A slightly superior mean body weight was evidenced for the two groups treated with the FE002 cell-laden hydrogels, as compared with the other treatment groups (Figure S7). Overall, the surgery and intra-articular injections were well tolerated by all animals. A slight body-weight-stabilization phase was observed across all groups after the intra-articular injection phase, due to the use of analgesic treatment (Figure S7). Several days post-surgery, in most of the cases, the operated knees were observed to be swollen. The presence of a subcutaneous mass was sometimes recorded on the thigh over the femur, appearing several days post-surgery. In all of the observed cases, the lump size spontaneously and progressively diminished, and the lump disappeared within four weeks post-OA induction. Such subcutaneous masses were probably due to an effusion of SF but did not affect the pathology development, as most of them were fully resorbed before the administration of the various combination products at day 63.

Upon terminal sampling at day 91 post-pathology-induction, local necropsy analysis was performed. No specific observations and no abnormalities were recorded. The presence of residual hematoma was observed only on one animal in the linear HA cell-laden group. The hematoma was localized outside the articular cavity, in the tissues surrounding the knee. In addition, a non-resorbable lump was observed on one animal of the HA-L-PNIPAM group. The lump presented a fibrotic aspect and was localized near the articular cavity.

**Figure 3:**
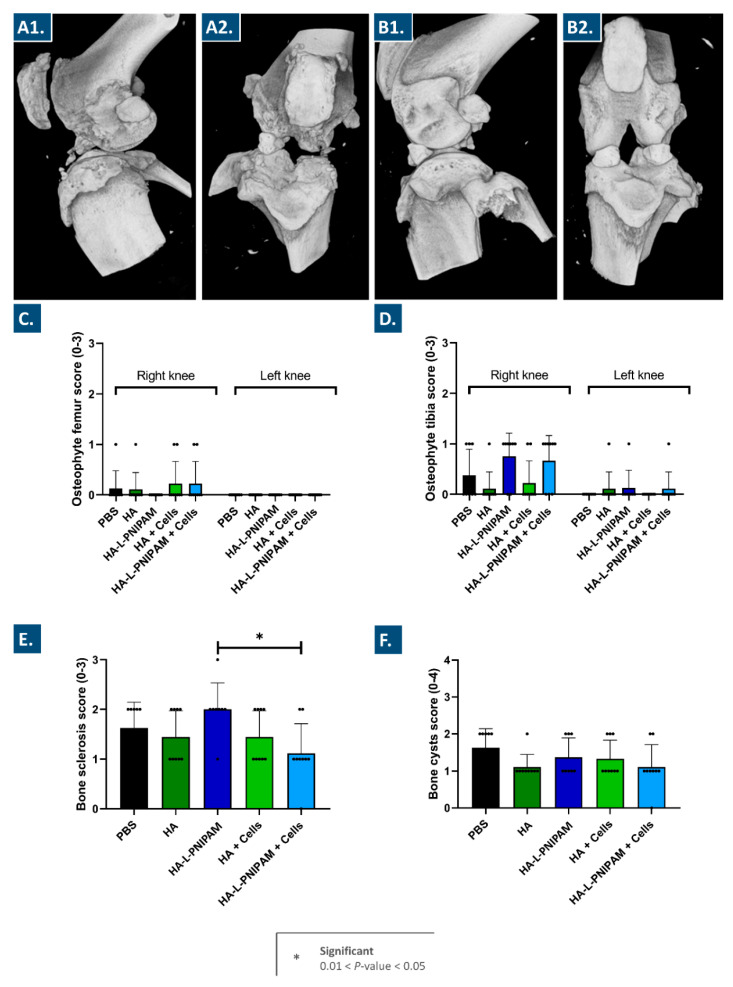
Illustrative 3D CT images of right knees (i.e., operated joints) (**A1**,**A2**) and left knees (i.e., non-operated joints) (**B1**,**B2**) from one animal of the treatment control group. Micro-CT images were blindly scored to evaluate osteophyte presence in the femur from right and left knees (**C**), osteophyte presence in the tibia from right and left knees (**D**), subchondral bone sclerosis in right knees (**E**), and bone cyst presence in right knees (**F**). Dot symbols represent individual scores per animal. Results are expressed as means with standard deviations as error bars for eight or nine animals per group. Significant statistical differences (i.e., * or 0.01 < *p*-value < 0.05) were found between presented mean values. HA, hyaluronic acid; PNIPAM, poly(N-isopropylacrylamide).

Following animal sacrifice, signs of bone lesions were visible on the available 3D images, with important differences observed between the right knees (i.e., operated joints, Figure 3A) and the contra-lateral left knees (i.e., non-operated joints, Figure 3B).

Transversal sections of each sample were prepared for the scoring of osteophyte and bone cyst presence (Figure S8, Tables S2–S4). The presence of subchondral bone sclerosis was scored on coronal images of the femurs and tibiae (Figure S9, Table S4). No significant differences were observed in osteophyte presence across all groups (Figure 3C,D). Interestingly, more osteophytes were observed in tibiae than in femurs, probably due to more mechanical compression applied on the tibial plateau. As regards bone sclerosis scoring, a statistically significant decrease in mean score values was outlined for the group treated with the HA-L-PNIPAM-based FE002 cell-laden hydrogels as compared to the group treated with the cell-free HA-L-PNIPAM-based hydrogels (Figure 3E). No other significant differences were found between the groups regarding bone sclerosis scoring. According to the literature, subchondral bone sclerosis is always observed in such animal models of OA, which was experimentally confirmed herein (Figure 3E) [32]. The bone cyst scores were found to be slightly lower in the groups treated with the combination products or with HA than in the control group treated with PBS (Figure 3F). These results suggest that the intra-articular injection of the HA-based or the HA-L-PNIPAM-based hydrogels with and without the lyophilized FE002 chondroprogenitors could potentially exert a beneficial effect on cyst formation.

Overall consideration of the scoring data obtained by the analysis of the micro-CT data enabled the assessment that the reported scores were relatively low, even within the control group (i.e., PBS injection in the osteoarthritic knee). This fact further contributed to validating the animal model choice and contributes to the methodological sturdiness of the adopted approach. Indeed, the choice of an animal model that did not comprise severe induction of knee OA was linked to established practices in human clinical orthopedics, wherein HA-based viscosupplementation is therapeutically applied in cases of mild to moderate knee OA.

**Figure 4:**
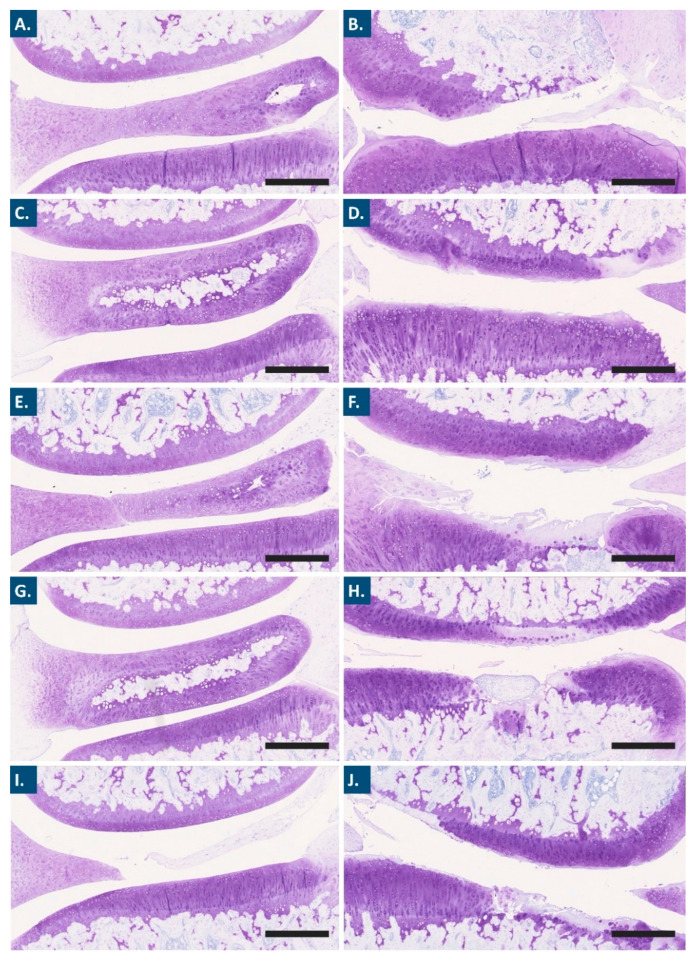
Representative Toluidine blue photomicrographs used for the grading of articular cartilage structure and the grading of proteoglycan contents. Photomicrographs showing the medial femoral condyle (above) and the medial tibial plateau (below) of non-operated left and operated right knees for the PBS (**A**,**B**), HA (**C**,**D**), HA-L-PNIPAM (**E**,**F**), HA + Cells (**G**,**H**), and HA-L-PNIPAM + Cells (**I**,**J**) groups, respectively. Scale bars = 500 µm. HA, hyaluronic acid; PNIPAM, poly(N-isopropylacrylamide).

**Figure 5:**
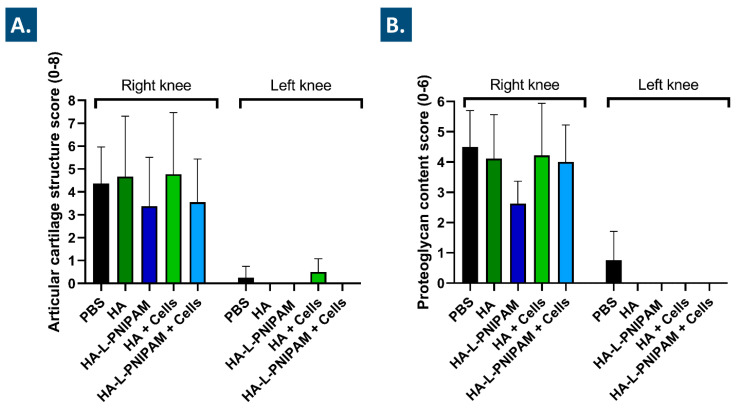
Medial tibial plateau semi-quantitative histological evaluation of articular cartilage structure (**A**) and proteoglycan content (**B**). Results are expressed as means with standard deviations as error bars for eight or nine animals per group. The OARSI scoring system was used for articular cartilage structure and proteoglycan content. HA, hyaluronic acid; PNIPAM, poly(N-isopropylacrylamide).

Further processing of the animal samples acquired post-sacrifice enabled the performance of extensive histological work. In particular, Mankin scoring of outcomes was applied for the semi-quantitative histopathologic evaluation of histological slides (Figure 4, Figure 5 and Figure S10) [57,60]. For each study group, microscopic analysis revealed a difference in articular cartilage morphology between the operated knees and the contra-lateral control knees. While the former presented moderate to marked OA lesions in the medial tibial plateau and medial femoral condyle, the latter only presented occasional surface irregularities (Figure 4).

A trend toward a decrease in the mean OARSI grades for the articular cartilage structure was observed for the HA-L-PNIPAM and the FE002 cell-laden HA-L-PNIPAM groups, as compared to the PBS control group (i.e., −23% and −18%, respectively, Figure 5A). In the groups treated with the linear HA-based combination products, no reductions in the mean OARSI grades were found for the articular cartilage structure (Figure 5A). Regarding proteoglycan contents and as compared to the PBS control group, a 42% decrease in the mean OARSI grades was observed in the HA-L-PNIPAM group. This decrease was found to be less important in amplitude for the FE002 cell-laden HA-L-PNIPAM group, with an 11% decrease in score values as compared to the PBS control group. In comparison, both linear HA-based formulations were associated with slight decreases of less than 10% in scoring values, as compared to the PBS control group.

Overall, the available experimental histological data enabled to conclude that clear and significant differences were obtained between the right and the left knees, confirming the appropriate and designed OA pathology induction after the ACLT-hMnx surgery. Therefore, HA-L-PNIPAM-based formulations tended to exert more protective effects than HA-based formulations, as demonstrated by the greater reduction in articular cartilage damage and loss of matrix (Figure 5).

**Figure 6:**
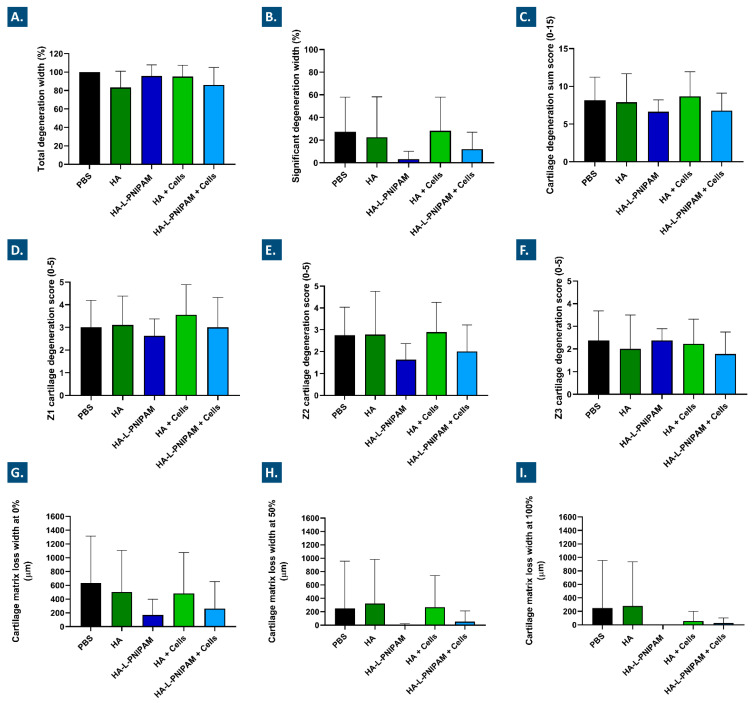
Medial tibial plateau histomorphometry evaluation of total (**A**) and significant (**B**) cartilage degeneration width. A percentage relative to the total width of the articular surface was used for total and significant cartilage degeneration width evaluation. A cartilage degeneration sum score for the medial tibial plateau was obtained by combining the scores of the three plateau sections (i.e., Z1 + Z2 + Z3) (**C**), considering the external third of the plateau (i.e., Z1) (**D**), the central third of the plateau (i.e., Z2) (**E**), and the internal third of the plateau (i.e., Z3) (**F**). Cartilage matrix loss width values were measured in the medial tibial plateau at 0% (i.e., at the surface, (**G**)), at 50% cartilage depth (**H**), and at 100% (**I**) cartilage depth, respectively. All results are expressed as means with standard deviations as error bars for eight or nine animals per group. HA, hyaluronic acid; PNIPAM, poly(N-isopropylacrylamide).

Further quantitative investigation (i.e., histomorphometry) into the evolution of chondral structures in the operated knees enabled adding granularity to the comparative assessment of the five treatment groups. In particular, the total cartilage degeneration width measurements showed very slight reduction values of −16%, −4%, −5%, and −14% for the HA, HA-L-PNIPAM, HA + Cells, and HA-L-PNIPAM + Cells groups, respectively (Figure 6A).

Nevertheless, a reduction in the severity of the OA lesions was shown for the cell-free and the cell-laden HA-L-PNIPAM groups, with decreases in the significant degeneration width of 83% and 55% in the medial tibial plateau, respectively (Figure 6B). Significant degeneration width is an important parameter that represents the width of the tibial cartilage in which 50% or more of the tissue thickness is seriously compromised [44]. In contrast, no clear reduction was outlined for significant cartilage degeneration in the medial tibial plateau for either of the linear HA-based formulations (Figure 6B). These results confirm the paramount importance of appropriate HA-based hydrogel selection for viscosupplementation product formulation, in order to exert a tangible effect on moderate knee OA.

The histomorphometric assessments performed on the harvested study materials also confirmed the presence of OA lesions in the medial tibial plateau of the operated knees within each group. No reduction in mean scores was observed for either of the linear HA groups (Figure 6C–F). Parallelly, slight decreases in the extension of the OA lesions for the cell-free HA-L-PNIPAM group and the cell-laden HA-L-PNIPAM group were observed, with 18% and 17% decreases in values, respectively (Figure 6C). Generally, it was found that the lesions were most extensive in the external third of the plateau (i.e., Z1, outside medial edge of the joint) within each group (Figure 6D). This result was expected, as the outer zone (Z1) is typically subjected to more mechanical constraints as compared to the central zones of the joint [61]. In the Z1 zone, only the cell-free HA-L-PNIPAM group showed a decrease in mean scores, which was 12% in value (Figure 6D). In the Z2 zone, both of the HA-L-PNIPAM-based formulations showed more important scoring decreases, with −48% in value for the cell-free HA-L-PNIPAM group and −27% in value for the cell-laden HA-L-PNIPAM group (Figure 6E). When considering scoring definitions, the results indicate that a mild degeneration of 11–25% affected both of the HA-L-PNIPAM groups, while a moderate degeneration of 26–50% affected the PBS control group and both of the HA groups.

Finally, the results for cartilage matrix loss width in the medial tibial plateau are presented in Figure 6G–I. The extension of the superficial OA lesions (i.e., the superficial cartilage matrix loss, at 0% depth) was similar within both of the HA groups, as compared to the PBS control group, and was congruent with the results obtained when considering the total degeneration width. The cartilage matrix loss at 0% depth indicated that there were fewer superficial fissures in the medial tibial plateau for the HA-L-PNIPAM groups (i.e., decreases of 61% and 40% in the cell-free HA-L-PNIPAM group and in the cell-laden HA-L-PNIPAM group, respectively, Figure 6G). At the level of the midzone (i.e., 50% depth), mean measurements of cartilage matrix loss were below 50 µm for both of the HA-L-PNIPAM formulations and equal or superior to 250 µm for all of the other groups (Figure 6H). No cartilage loss was evidenced in the deep zones (i.e., 100% depth) for the HA-L-PNIPAM group, while mean loss widths of 26 µm and 56 µm were reported for the FE002 cell-laden HA-L-PNIPAM group and for the FE002 cell-laden HA group, respectively (Figure 6I).

Aggregation of the available in vivo data has shown that, whereas significant differences were found between the groups in terms of bone sclerosis and cysts, no significant differential effects were observed in terms of histomorphometry for cartilage degeneration scores and width (Figure 3, Figure 5 and Figure 6). For instance, although the effects of reduction on cartilage matrix loss were found to be important (i.e., >3-fold reduction for HA-L-PNIPAM versus PBS), they were not considered to be significant due to the high data variability (Figure 6G–I). The latter was linked to the intrinsic variability of the ACLT-hMnx OA model, as well as to the number (i.e., n = 8–9/group) of animals selected for the in vivo study. As regards product efficacy demonstration, the presented results would indeed warrant further studies in more controlled OA models with larger groups, yet the significance of such models in terms of translational and clinical relevance is difficult to validate, as discussed in the following section. Overall, the setting and the objective of the in vivo part of the study must be taken into consideration during data interpretation. Specifically, the compiled work presented herein has confirmed the applicability and the safety of the considered interventions, which constitutes a tangible milestone.

Indeed, comprehensive consideration of the experimental data from the animal study enabled concluding that the combination products containing HA-L-PNIPAM and the stabilized FE002 chondroprogenitors did not cause significant adverse reactions in vivo and procured beneficial trends in a rodent model of knee OA, as compared to linear HA-based combination products and PBS controls. Specifically, the validity, adequacy, and usefulness of the retained knee OA model were all confirmed for this study. Based on such results and insights, further studies on combination product efficacy may be devised, with appropriate adaptations to the retained model or preclinical setting, the product parameters, the product administration regimen, and the study statistical power.

### 3.8. Novel Orthopedic Combination Product Preclinical Assessment: Current Insights and Bottlenecks around Efficacy Evaluation

It is noteworthy that recent scientific literature and regulatory analyses have outlined a relative scarcity and inhomogeneity in the existing preclinical data submitted within market approval procedures for cell- or gene-based orthopedic products [62]. Specifically, it was reported that out of 23 FDA-authorized cell and gene therapy products, no preclinical GLP-compliant study had been performed on animal models, and that many costly animal models have been used excessively without much efficacy-related relevance for OA applications in particular [62]. The results presented herein for the rat ACLT-hMnx OA model fall into alignment with similar conclusions recently drawn in the orthopedic field, as no safety concerns were evidenced for any of the injected formulas. However, based on the retained endpoints and readouts for the rat ACLT-hMnx model, functional or efficacy-related data were found to be tendential at best (Figure 3, Figure 5, and Figure 6). However, such results should be put into perspective from an efficacy point of view, as products similar (i.e., rheology-wise, e.g., Ostenil, TRB Chemedica, Switzerland) to the cell-free HA-based formula used herein (i.e., assessed as non-different from PBS controls in histology, Figure 5 and Figure 6) are routinely and successfully used in clinical practice for viscosupplementation applications [17,63,64,65,66,67,68,69]. Furthermore, it appears evident that many of the patient-reported scored outcomes widely used in human medicine are inapplicable in animal models of OA, which does not help to bridge the divide between preclinical efficacy evaluation and clinical efficacy studies. Such considerations and the reported lack of strong methodological consensus should prompt further optimization and rationalization of animal model use, as the direct benefit to orthopedic patients in particular (i.e., by means of treatment with approved novel products) is increasingly difficult to demonstrate [62].

Furthermore, the assessment of novel orthopedic cell-based products is challenging with regard to the elucidation of the mechanisms of action or the appropriate efficacy attributes of the product [62]. Indeed, while several biologically-based combination therapies have been successfully used (e.g., autologous PRP-laden HA hydrogels) in knee OA patients, the majority of the available efficacy-related data comes from human clinical practice and patient-reported results, not from animal studies [28,33]. Preclinical animal work is currently still required by regulators in most cases of novel product development, notably for the demonstration of safety before moving to phase I human clinical trials. However, due to the increasing public pressure to limit animal work to strict minima, tangible alternatives need to be identified by scholars and industrial manufacturers, in order to ensure the continuity in safe and effective novel product development and commercialization [62].

### 3.9. A Historical Landmark in Clinical Allogeneic Cell-Based Management of Orthopedic Conditions: The Disruptive Case of Invossa

A prime example of a clinically investigated allogeneic cell-based solution for the management of orthopedic conditions is the bi-component cytotherapeutic kit known as Invossa (Kolon TissueGene, Rockville, MA, USA), which is discussed herein to contextualize the proposed use of allogeneic FE002 chondroprogenitors for cartilage regenerative medicine [42,70]. The Invossa product, a first-in-class orthopedic cell and gene therapy product, is composed of a mixture of cell populations (i.e., allogeneic non-transformed polydactyly chondrocytes and cryopreserved, irradiated, and TGF-β-transfected HEK-293 viable cells, i.e., immortalized fetal kidney-derived cells) [70,71,72]. Curiously, Invossa was supposed to contain retrovirally transduced human chondrocytes expressing TGF-β instead of HEK-293 cells [70,73,74]. Following an apparent vial mislabeling and the treatment of large patient collectives with preparations containing HEK-293 cells, the clinical trials for Invossa were interrupted. However, interestingly, phase 3 clinical work has resumed in the United States based on the demonstrated safety of the HEK-293 cells and on the recorded pain reduction efficacy in the available clinical human data (i.e., absence of adverse event occurrence, meeting of efficacy endpoints) [70,74]. The approved and ongoing investigational use (i.e., multi-centric clinical trials) of Invossa in the USA creates a strong precedent for the application of prenatal tissue-derived cytotherapeutics in human regenerative medicine [70,74]. Several technical elements require further clarification in the case of Invossa (e.g., potential cross-contaminations by HEK-293 substrates during cell manufacturing), yet the specified orthopedic clinical endpoints of the intervention were reported to have been met nonetheless [74]. Although the case of Invossa constitutes an exception and not the norm, it is understood that while robust preclinical data is necessary for the exhaustive understanding of a specific new therapeutic product, human clinical reports have the most impact regulatory-wise and economy-wise. This is illustrated by the use of the safety data initially generated around Invossa (i.e., prior to the identification of the mislabeling issue) to convince regulators to authorize re-initiation of the investigative clinical work. Finally, the fact that immortalized fetal cells (i.e., in Invossa) have been successfully used in practice at large orthopedic clinical scales represents a strong precedent for the use of primary chondroprogenitors as described herein, with further potential for relatively enhanced product standardization and off-the-shelf formulation. 

### 3.10. Study Limitations and Perspectives for Future Development Work

The main study limitations in terms of combination product characterization resided in the limited presented scope of the experimental data (e.g., absence of further component compatibility study, combination product long term stability assays). However, the focus of the presented work was set by design on the functional aspects of the considered FE002 cellular materials and combination products, which constitute essential attributes for product development and manufacturing control [43]. Therefore, based on the methodological aspects presented herein, it may be possible to validate further exhaustive characterization work on given prototypes, based on the fact that they may be produced in a scalable manner while retaining high quality and function. Such approaches may be considered for rational and functionality-driven product or device development, coupled with appropriate in vivo readouts and endpoints within the full multifactorial complexity of joint OA.

Based notably on the original in vivo data presented herein, several future areas of focus have been identified, such as the further investigation of FE002 cell-based cell-free preparations, as well as further preclinical assessments of injectable orthopedic combination products. Namely, while the retained rat OA model was validated and assessed as optimal for the needs of the in vivo work presented herein, further adaptation of the model (i.e., earlier intra-articular treatment following the ACLT-hMnx surgery) or the use of an alternative therapeutic posology (e.g., repeated product administration, as clinically applied in human medicine) could potentially be beneficial in the context of preclinical combination product efficacy assessment [75]. Overall, further translational work shall be designed to take into account the clinical pathways followed by human OA patients, in order to match the attributes and functions of the proposed products to the existing clinical and medical needs.

## 4. Conclusions

Throughout the various combination product functional qualification assays presented in this study, notable advantages were outlined for the use of HA-L-PNIPAM over linear HA in the formulation of injectable cytotherapeutics for mild to moderate knee OA management. It was shown that the considered combination products were well adapted to the intended knee OA application from a preclinical translational point-of-view (i.e., internal compatibility, possibility to autoclave-sterilize the hydrogels, cytocompatibility, injectability). Specifically, the combination formulas containing HA-L-PNIPAM displayed improved viscoelastic behaviors (i.e., significant rise in G′ and G″ at body temperature), lubrication abilities, and resistance toward enzymatic or oxidative degradation as compared to native linear hydrogels (i.e., conventional HA-based formulations). While the considered lyophilized FE002 chondroprogenitors presented significant intrinsic antioxidant properties, the key functionalities thereof within the combination products (i.e., from a mechanistic point-of-view) were identified in vitro as being potent hydrogel functionalizing agents for improved stability against oxidative damages. Furthermore, extensive multi-parametric in vivo investigation of the effects of FE002 cell-laden HA-L-PNIPAM in an ACLT-hMnx rat model of knee OA revealed no general or local adverse effects, as well as mild to moderate beneficial trends (i.e., histological scoring, histomorphometry analyses) against the development of chondral defects. The aspects of product cytocompatibility with human cells, biocompatibility in a rodent model, and appropriate syringeability have laid the technical basis for further translational preclinical work. Overall, the presented study addressed key aspects of novel orthopedic cytotherapeutic product preclinical development and yielded encouraging results on combinational approaches for knee OA clinical management.

## Figures and Tables

**Table 1:** Summary of the various considered combination product formulations used for the in vitro and in vivo characterization experiments. The HA raw materials were obtained from the same source for each group (i.e., 2.2–2.4 MDa MW ^1^), and each formulation was submitted to the same sterilization cycle before direct use or incorporation of the lyophilized FE002 cells. Therefore, all characterization experiments were performed using steam-sterilized hydrogels. HA, hyaluronic acid; MW, molecular weight; PNIPAM, poly(N-isopropylacrylamide).

Formulation Type	Gross (Co-)Polymer Concentration (mg/mL)	Net HA Concentration (mg/mL) ^2^	Lyophilized FE002 Chondroprogenitor Presence (Y/N)
HA	10	10	N
HA-L-PNIPAM	37	10	N
HA + Cells	10	10	Y (3 × 10^6^ cells/vial)
HA-L-PNIPAM + Cells	37	10	Y (3 × 10^6^ cells/vial)

^1^ The retained HA raw material specifications (i.e., molecular weight class) were selected based on technical imperatives (i.e., need of easy injection, maximization of post-sterilization rheological properties) and with focus on clinical/functional aspects (i.e., maximization of in situ product viscosity and residence time, for enhanced function). ^2^ The net HA contents for the HA-L-PNIPAM co-polymers were calculated according to the degrees of substitution experimentally obtained in ^1^H NMR spectroscopy characterization.

## Data Availability

The data presented in this study are available upon reasonable request made to the corresponding authors. The data are not publicly available due to legal and statutory restrictions.

## Supplementary Materials

The following supporting information can be downloaded at https://www.mdpi.com/article/10.3390/pharmaceutics15051528/s1. Figure S1: Synthetic routes of HA-L-PNIPAM; Figure S2: Analytical controls of HA-L-PNIPAM synthesis; Figure S3: Effects of steam sterilization on the attributes of HA-based hydrogels; Figure S4: Results of the WST-1 cytotoxicity assay; Figure S5: Results of the hydrogel injectability assays; Figure S6: Animal study general workflow; Figure S7: Body weight curves for the animal study; Figure S8: Representative imaging for CT-scan data; Figure S9: Representative imaging for CT-scan data; Figure S10: Representative imaging for histological data; Table S1: Grading results within the FE002 chondroprogenitor lyophilization phase; Table S2: Osteophyte scoring table; Table S3: Bone cyst scoring table; Table S4: Bone sclerosis scoring table; Table S5: Articular cartilage structure and proteoglycan content scoring table.

## Abbreviations

ACLT	anterior cruciate ligament transection
Da	Daltons
HA	hyaluronic acid
H_2_O_2_	hydrogen peroxide
FRAP	ferric reducing antioxidant power
GLP	good laboratory practices
HES	hematoxilin/eosin/saffron
hMnx	hemi-meniscectomy
LVE	linear viscoelastic region
MD	medical device
MW	molecular weight
OA	osteoarthritis
OARSI	Osteoarthritis Research Society International
Pa	Pascals
Pa·s	Pascal seconds
PBS	phosphate-buffered saline
Ph.Eur.	European pharmacopoeia
PNIPAM	poly(N-isopropylacrylamide)
PRP	platelet-rich plasma
ROS	reactive oxygen species
SF	synovial fluid
TB	Toluidine blue
TEAC	Trolox equivalent antioxidant capacity
USA	United States of America
UV	ultraviolet

## References

[1] Martell-Pelletier J., Pelletier J.P., Lajeunesse D., Koopman W.J. (2005). Etiopathogenesis of osteoarthritis. Arthritis and Allied Conditions—A Textbook of Rheumatology.

[2] Primorac D., Molnar V., Rod E., Jeleč Ž., Čukelj F., Matišić V., Vrdoljak T., Hudetz D., Hajsok H., Borić I. (2020). Knee osteoarthritis: A review of pathogenesis and state-of-the-art non-operative therapeutic considerations. Genes.

[3] Martel-Pelletier J., Barr A.J., Cicuttini F.M., Conaghan P.G., Cooper C., Goldring M.B., Goldring S.R., Jones G., Teichtahl A.J., Pelletier J.P. (2016). Osteoarthritis. Nat. Rev. Dis. Primers.

[4] Loeser R.F., Goldring S.R., Scanzello C.R., Goldring M.B. (2012). Osteoarthritis: A disease of the joint as an organ. Arthritis Rheumatol..

[5] Malekipour F., Lee P.V. (2019). Shock absorbing ability in healthy and damaged cartilage-bone under high-rate compression. J. Mech. Behav. Biomed. Mat..

[6] Tamer T.M. (2013). Hyaluronan and synovial joint: Function, distribution and healing. Interdiscipl. Toxicol..

[7] Schmidt T.A., Sah R.L. (2007). Effect of synovial fluid on boundary lubrication of articular cartilage. Osteoarthr. Cartil..

[8] Nurul A.A., Azlan M., Ahmad Mohd Zain M.R., Sebastian A.A., Fan Y.Z., Fauzi M.B. (2021). Mesenchymal stem cells: Current concepts in the management of inflammation in osteoarthritis. Biomedicines.

[9] Arden N.K., Perry T.A., Bannuru R.R., Bruyère O., Cooper C., Haugen I.K., Hochberg M.C., McAlindon T.E., Mobasheri A., Reginster J.Y. (2021). Non-surgical management of knee osteoarthritis: Comparison of ESCEO and OARSI 2019 guidelines. Nat. Rev. Rheumatol..

[10] Jones I.A., Togashi R., Wilson M.L., Heckmann N., Vangsness C.T. (2019). Intra-articular treatment options for knee osteoarthritis. Nat. Rev. Rheumatol..

[11] Rzhepakovsky I., Anusha Siddiqui S., Avanesyan S., Benlidayi M., Dhingra K., Dolgalev A., Enukashvily N., Fritsch T., Heinz V., Kochergin S. (2021). Anti-arthritic effect of chicken embryo tissue hydrolyzate against adjuvant arthritis in rats (X-ray microtomographic and histopathological analysis). Food Sci. Nutr..

[12] Zhao J.M., Chen X., Cheng K., Shi Q., Peng K. (2020). Anserine and glucosamine supplementation attenuates the levels of inflammatory markers in rats with rheumatoid arthritis. AMB Express.

[13] Plaas A.H.K., Moran M.M., Sandy J.D., Hascall V.C. (2023). Aggrecan and hyaluronan: The infamous cartilage polyelectrolytes—Then and now. Adv. Exp. Med. Biol..

[14] Porcello A., Gonzalez-Fernandez P., Jordan O., Allémann E. (2022). Nanoforming hyaluronan-based thermoresponsive hydrogels: Optimized and tunable functionality in osteoarthritis management. Pharmaceutics.

[15] Testa G., Giardina S.M.C., Culmone A., Vescio A., Turchetta M., Cannavò S., Pavone V. (2021). Intra-articular injections in knee osteoarthritis: A review of literature. J. Funct. Morphol. Kinesiol..

[16] Xing D., Wang B., Liu Q., Ke Y., Xu Y., Li Z., Lin J. (2016). Intra-articular hyaluronic acid in treating knee osteoarthritis: A PRISMA-compliant systematic review of overlapping meta-analysis. Sci. Rep..

[17] Pereira T.V., Jüni P., Saadat P., Xing D., Yao L., Bobos P., Agarwal A., Hincapié C.A., da Costa B.R. (2022). Viscosupplementation for knee osteoarthritis: Systematic review and meta-analysis. BMJ.

[18] Ayhan E., Kesmezacar H., Akgun I. (2014). Intraarticular injections (corticosteroid, hyaluronic acid, platelet rich plasma) for the knee osteoarthritis. World J. Orthop..

[19] Juhaščik M., Kováčik A., Huerta-Ángeles G. (2022). Recent advances of hyaluronan for skin delivery: From structure to fabrication strategies and applications. Polymers.

[20] Conrozier T., Mathieu P., Rinaudo M. (2014). Mannitol preserves the viscoelastic properties of hyaluronic acid in an in vitro model of oxidative stress. Rheumatol. Ther..

[21] Cao Y., Ma Y., Tao Y., Lin W., Wang P. (2021). Intra-articular drug delivery for osteoarthritis treatment. Pharmaceutics.

[22] Larsen C., Ostergaard J., Larsen S.W., Jensen H., Jacobsen S., Lindegaard C., Andersen P.H. (2008). Intra-articular depot formulation principles: Role in the management of postoperative pain and arthritic disorders. J. Pharm. Sci..

[23] Žádníková P., Šínová R., Pavlík V., Šimek M., Šafránková B., Hermannová M., Nešporová K., Velebný V. (2022). The degradation of hyaluronan in the skin. Biomolecules.

[24] Laurent A., Porcello A., Fernandez P.G., Jeannerat A., Peneveyre C., Abdel-Sayed P., Scaletta C., Hirt-Burri N., Michetti M., de Buys Roessingh A. (2021). Combination of hyaluronan and lyophilized progenitor cell derivatives: Stabilization of functional hydrogel products for therapeutic management of tendinous tissue disorders. Pharmaceutics.

[25] Hintze V., Schnabelrauch M., Rother S. (2022). Chemical modification of hyaluronan and their biomedical applications. Front. Chem..

[26] Schanté C., Zubera G., Herlinb C., Vandammea T.F. (2011). Chemical modifications of hyaluronic acid for the synthesis of derivatives for a broad range of biomedical applications. Carbohydr. Polym..

[27] Maudens P., Meyer S., Seemayer C.A., Jordan O., Allémann E. (2018). Self-assembled thermoresponsive nanostructures of hyaluronic acid conjugates for osteoarthritis therapy. Nanoscale.

[28] Zhou T., Ran J., Xu P., Shen L., He Y., Ye J., Wu L., Gao C. (2022). A hyaluronic acid/platelet-rich plasma hydrogel containing MnO_2_ nanozymes efficiently alleviates osteoarthritis in vivo. Carbohydr. Polym..

[29] Li J., Zhang H., Han Y., Hu Y., Geng Z., Su J. (2023). Targeted and responsive biomaterials in osteoarthritis. Theranostics.

[30] Aguilar M.R., Roman J.S., Aguilar M.R., Roman J.S. (2019). Introduction to smart polymers and their applications. Smart Polymers and Their Applications.

[31] Sponchioni M., Palmiero U.C., Moscatelli D. (2019). Thermo-responsive polymers: Applications of smart materials in drug delivery and tissue engineering. Mat. Sci. Eng. C.

[32] Chiang E.R., Ma H.L., Wang J.P., Liu C.L., Chen T.H., Hung S.C. (2016). Allogeneic mesenchymal stem cells in combination with hyaluronic acid for the treatment of osteoarthritis in rabbits. PLoS ONE.

[33] Yu W., Xu P., Huang G., Liu L. (2018). Clinical therapy of hyaluronic acid combined with platelet-rich plasma for the treatment of knee osteoarthritis. Exp. Ther. Med..

[34] López-Ruiz E., Jiménez G., Álvarez de Cienfuegos L., Antic C., Sabata R., Marchal J.A., Gálvez-Martín P. (2019). Advances of hyaluronic acid in stem cell therapy and tissue engineering, including current clinical trials. Eur. Cells Mater..

[35] Li L., Duan X., Fan Z., Chen L., Xing F., Xu Z., Chen Q., Xiang Z. (2018). Mesenchymal stem cells in combination with hyaluronic acid for articular cartilage defects. Sci. Rep..

[36] Wang Y., Yu D., Liu Z., Zhou F., Dai J., Wu B., Zhou J., Heng B.C., Zou X.H., Ouyang H. (2017). Exosomes from embryonic mesenchymal stem cells alleviate osteoarthritis through balancing synthesis and degradation of cartilage extracellular matrix. Stem Cell Res. Ther..

[37] Jin Y., Xu M., Zhu H., Dong C., Ji J., Liu Y., Deng A., Gu Z. (2021). Therapeutic effects of bone marrow mesenchymal stem cells-derived exosomes on osteoarthritis. J. Cell. Mol. Med..

[38] Chen W., Sun Y., Gu X., Hao Y., Liu X., Lin J., Chen J., Chen S. (2019). Conditioned medium of mesenchymal stem cells delays osteoarthritis progression in a rat model by protecting subchondral bone, maintaining matrix homeostasis, and enhancing autophagy. J. Tissue Eng. Regen. Med..

[39] Zhao H., Zhao Z., Li D., Wang X., Dai D., Fu H. (2023). Effect study of exosomes derived from platelet-rich plasma in the treatment of knee cartilage defects in rats. J. Orthop. Surg. Res..

[40] Liu Y., Zeng Y., Si H.B., Tang L., Xie H.Q., Shen B. (2022). Exosomes derived from human urine-derived stem cells overexpressing miR-140-5p alleviate knee osteoarthritis through downregulation of VEGFA in a rat model. Am. J. Sports Med..

[41] Huang G.S., Peng Y.J., Hwang D.W., Lee H.S., Chang Y.C., Chiang S.W., Hsu Y.C., Liu Y.C., Lin M.H., Wang C.Y. (2021). Assessment of the efficacy of intra-articular platelet rich plasma treatment in an ACLT experimental model by dynamic contrast enhancement MRI of knee subchondral bone marrow and MRI T2∗ measurement of articular cartilage. Osteoarthr. Cartil..

[42] Laurent A., Abdel-Sayed P., Ducrot A., Hirt-Burri N., Scaletta C., Jaccoud S., Nuss K., Roessingh A., Raffoul W., Pioletti D. (2021). Development of standardized fetal progenitor cell therapy for cartilage regenerative medicine: Industrial transposition and preliminary safety in xenogeneic transplantation. Biomolecules.

[43] Laurent A., Porcello A., Jeannerat A., Peneveyre C., Coeur A., Abdel-Sayed P., Scaletta C., Michetti M., de Buys Roessingh A., Jordan O. (2023). Lyophilized progenitor tenocyte extracts: Sterilizable cytotherapeutic derivatives with antioxidant properties and hyaluronan hydrogel functionalization effects. Antioxidants.

[44] Gerwin N., Bendele A.M., Glasson S., Carlson C.S. (2010). The OARSI histopathology initiative—Recommendations for histological assessments of osteoarthritis in the rat. Osteoarthr. Cartil..

[45] Teeple E., Jay G.D., Elsaid K.A., Fleming B.C. (2013). Animal models of osteoarthritis: Challenges of model selection and analysis. AAPS J..

[46] Ginesin E., Chari N.S., Barnhart J., Wojnowski N., Patel R.M. (2023). Cartilage restoration for isolated patellar chondral defects: An updated systematic review. Orthop. J. Sports Med..

[47] Laurent A., Scaletta C., Abdel-Sayed P., Michetti M., Flahaut M., Simon J.P., Roessingh A.B., Raffoul W., Hirt-Burri N., Applegate L.A. (2021). Optimized manufacture of lyophilized dermal fibroblasts for next-generation off-the-shelf progenitor biological bandages in topical post-burn regenerative medicine. Biomedicines.

[48] Hunsberger J., Harrysson O., Shirwaiker R., Starly B., Wysk R., Cohen P., Allickson J., Yoo J., Atala A. (2015). Manufacturing road map for tissue engineering and regenerative medicine technologies. Stem Cells Transl. Med..

[49] Philippe V., Laurent A., Hirt-Burri N., Abdel-Sayed P., Scaletta C., Schneebeli V., Michetti M., Brunet J.F., Applegate L.A., Martin R. (2022). Retrospective analysis of autologous chondrocyte-based cytotherapy production for clinical use: GMP process-based manufacturing optimization in a Swiss university hospital. Cells.

[50] Lázaro B., Alonso P., Rodriguez A., La Nuez M., Marzo F., Prieto J.G. (2018). Characterization of the visco-elastic properties of hyaluronic acid. Biorheology.

[51] Nicholls M., Manjoo A., Shaw P., Niazi F., Rosen J. (2018). A comparison between rheological properties of intra-articular hyaluronic acid preparations and reported human synovial fluid. Adv. Ther..

[52] Nicholls M., Manjoo A., Shaw P., Niazi F., Rosen J. (2018). Rheological properties of commercially available hyaluronic acid products in the United States for the treatment of osteoarthritis knee pain. Clin. Med. Insights Arthritis Musculoskelet. Disord..

[53] Bonnevie E.D., Galesso D., Secchieri C., Bonassar L.J. (2019). Frictional characterization of injectable hyaluronic acids is more predictive of clinical outcomes than traditional rheological or viscoelastic characterization. PLoS ONE.

[54] Schanté C., Zuber G., Herlin C., Vandamme T.F. (2011). Synthesis of N-alanyl-hyaluronamide with high degree of substitution for enhanced resistance to hyaluronidase-mediated digestion. Carbohydr. Polym..

[55] Cilurzo F., Selmin F., Minghetti P., Adami M., Bertoni E., Lauria S., Montanari L. (2011). Injectability evaluation: An open issue. AAPS PharmSciTech.

[56] Robinson T.E., Hugues E.A., Bose A., Cornish E.A., Teo J.Y., Eisenstein E.M., Grover L.M., Cox S.C. (2020). Filling the gap: A correlation between objective and subjective measures of injectability. Adv. Health Mater..

[57] Cope P.J., Ourradi K., Li Y., Sharif M. (2019). Models of osteoarthritis: The good, the bad and the promising. Osteoarthr. Cartil..

[58] Pickarski M., Hayami T., Zhuo Y., Duong L.T. (2011). Molecular changes in articular cartilage and subchondral bone in the rat anterior cruciate ligament transection and meniscectomized models of osteoarthritis. BMC Musculoskelet. Disord..

[59] Kuyinu E.L., Narayanan G., Nair L.S., Laurencin C.T. (2016). Animal models of osteoarthritis: Classification, update, and measurement of outcomes. J. Orthop. Surg. Res..

[60] McCoy A.M. (2015). Animal models of osteoarthritis: Comparisons and key considerations. Vet. Pathol..

[61] Nielsen R.H., Bay-Jensen A.C., Byrjalsen I., Karsdal M.A. (2011). Oral salmon calcitonin reduces cartilage and bone pathology in an osteoarthritis rat model with increased subchondral bone turnover. Osteoarthr. Cartil..

[62] Nordberg R.C., Otarola G.A., Wang D., Hu J.C., Athanasiou K.A. (2022). Navigating regulatory pathways for translation of biologic cartilage repair products. Sci. Transl. Med..

[63] Tikiz C., Unlü Z., Sener A., Efe M., Tüzün C. (2005). Comparison of the efficacy of lower and higher molecular weight viscosupplementation in the treatment of hip osteoarthritis. Clin. Rheumatol..

[64] Blicharski T., Łukasik P., Plebanski R., Żęgota Z., Szuścik M., Moster E., Pavelka K., Jeon S., Park S. (2023). Efficacy and safety of intra-articular cross-linked sodium hyaluronate for the treatment of knee osteoarthritis: A prospective, active-controlled, randomized, parallel-group, double-blind, multicenter study. J. Clin. Med..

[65] Vincent P. (2021). Intra-articular hyaluronic acid in knee osteoarthritis: Clinical data for a product family (ARTHRUM), with comparative meta-analyses. Curr. Ther. Res. Clin. Exp..

[66] Baron D., Flin C., Porterie J., Despaux J., Vincent P. (2018). Hyaluronic acid single intra-articular injection in knee osteoarthritis: A multicenter open prospective study (ART-ONE 75) with placebo post hoc comparison. Curr. Ther. Res. Clin. Exp..

[67] Vincent P., Lucas de Couville T., Thomas T. (2020). Intra-articular hyaluronic acid for knee osteoarthritis: A postmarket, open-label, long-term historical control study with analysis detailed per Krellgren-Lawrence radiologic osteoarthritis scale grade. Curr. Ther. Res. Clin. Exp..

[68] Chavda S., Rabbani S.A., Wadhwa T. (2022). Role and effectiveness of intra-articular injection of hyaluronic acid in the treatment of knee osteoarthritis: A systematic review. Cureus.

[69] Altman R., Hackel J., Niazi F., Shaw P., Nicholls M. (2018). Efficacy and safety of repeated courses of hyaluronic acid injections for knee osteoarthritis: A systematic review. Semin. Arthritis Rheum..

[70] Evans C.H., Ghivizzani S.C., Robbins P.D. (2021). Orthopaedic gene therapy: Twenty-five years on. JBJS Rev..

[71] Noh M.J., Copeland R.O., Yi Y., Choi K.B., Meschter C., Hwang S., Lim C.L., Yip V., Hyun J.P., Lee H.Y. (2010). Pre-clinical studies of retrovirally transduced human chondrocytes expressing transforming growth factor-beta-1 (TG-C). Cytotherapy.

[72] Lee B. (2018). INVOSSA, a first-in-class of cell and gene therapy for osteoarthritis treatment: The phase III trial. Osteoarthr. Cartil..

[73] Ha C.W., Noh M.J., Choi K.B., Lee K.H. (2012). Initial phase I safety of retrovirally transduced human chondrocytes expressing transforming growth factor-beta-1 in degenerative arthritis patients. Cytotherapy.

[74] Kim M.K., Ha C.W., In Y., Cho S.D., Choi E.S., Ha J.K., Lee J.H., Yoo J.D., Bin S.I., Choi C.H. (2018). A multicenter, double-blind, phase III clinical trial to evaluate the efficacy and safety of a cell and gene therapy in knee osteoarthritis patients. Hum. Gene Ther..

[75] Peck J., Slovek A., Miro P., Vij N., Traube B., Lee C., Berger A.A., Kassem H., Kaye A.D., Sherman W.F. (2021). A comprehensive review of viscosupplementation in osteoarthritis of the knee. Orthop. Rev..

[76] World Medical Association (2013). World Medical Association Declaration of Helsinki: Ethical principles for medical research involving human subjects. JAMA.

