# Supplementary material for: Thermo-Responsive Hyaluronan-Based Hydrogels Combined with Allogeneic Cytotherapeutics for the Treatment of Osteoarthritis
Source: Pharmaceutics. 2023 May 18;15(5):1528. doi: 10.3390/pharmaceutics15051528 (PMC10224378; doi:10.3390/pharmaceutics15051528)
Supplement: Supplementary file 1 [file pharmaceutics-15-01528-s001.zip › pharmaceutics-2400595-supplementary.pdf]

Supplementary Figures

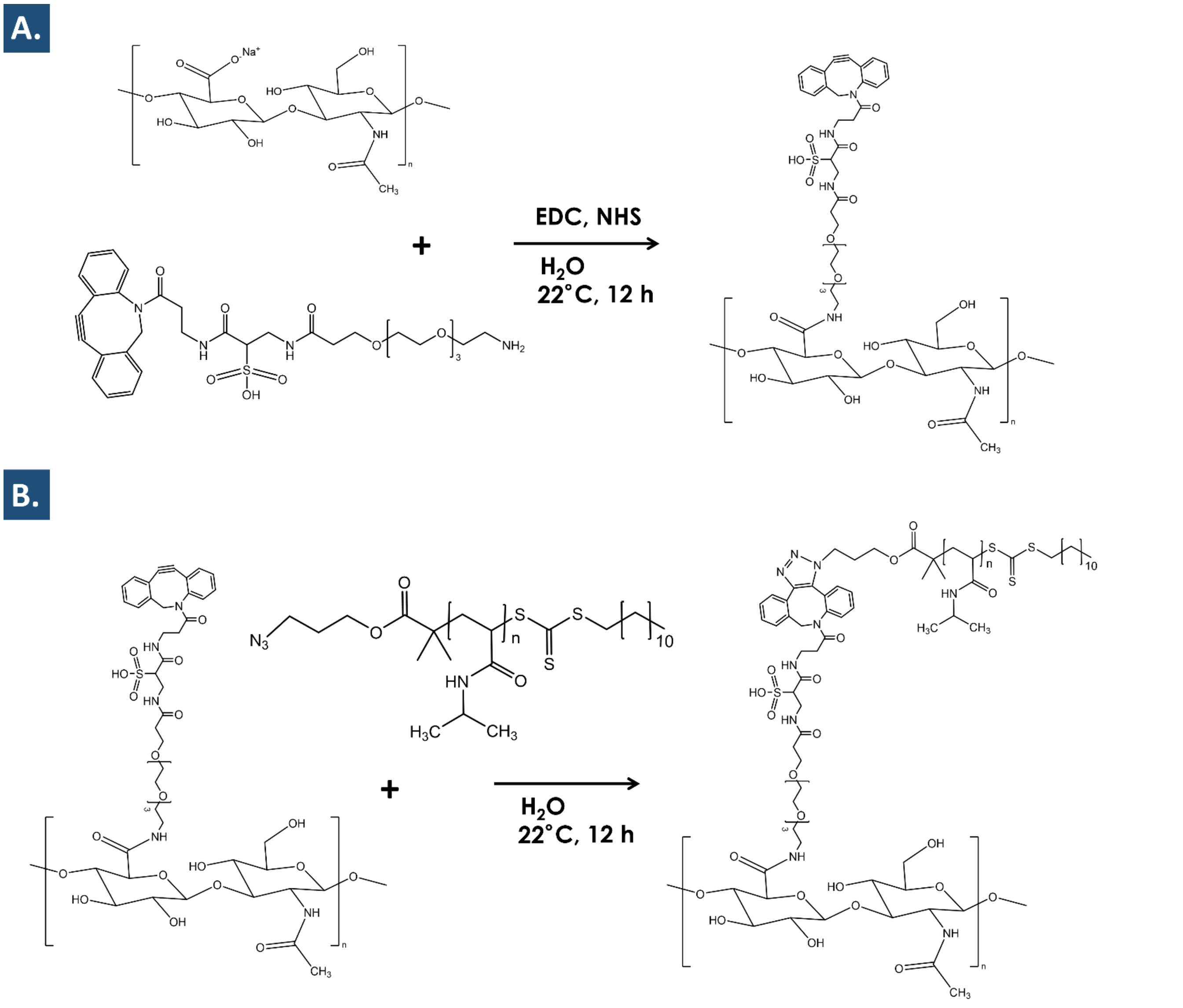

Figure S1. Synthetic routes of HA-L-PNIPAM. Amidation reaction (**A**) and copper-free azide-DBCO click chemistry reaction (**B**). EDC, ethylene dichloride; HA, hyaluronic acid; NHS, n-hydroxysuccinimide; PNIPAM, poly(N-isopropylacrylamide).

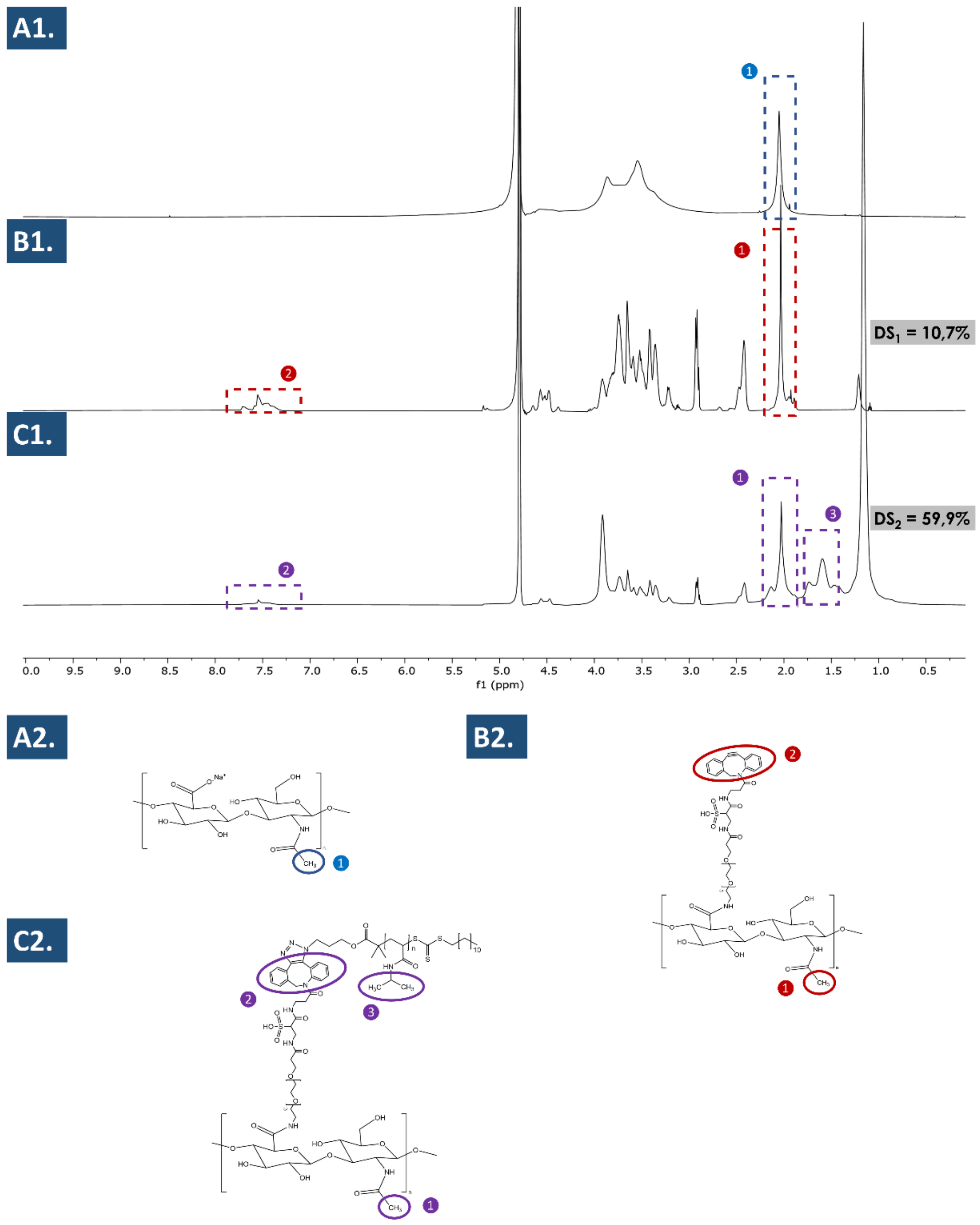

Figure S2. Characterization data for HA-L-PNIPAM and synthesis intermediates, with ^{1}H-NMR spectra in D_2O of HA (**A1** and **A2**), HA-sulfo-DBCO-PEG4 with the DS_1 (**B1** and **B2**), and HA-L-PNIPAM 0.5 eq. with the DS_2 (**C1** and **C2**). For the obtention of the spectra presented under **B** and **C**, the polymers were submitted to hyaluronidase before NMR analysis. The specific assignments of the proton NMR resonance peaks were δ = 2.0 ppm (**1**), 4.7–2.7 ppm (HA-backbone/PEG-DBCO), 7.2–7.7 ppm (**2**), and 1.6 ppm (**3**). Experimental mean DS_1 values (HA-sulfo-DBCO-PEG4) were of 10.7%. Experimental mean DS_2 values (HA-L-PNIPAM) were of 59.9%. According to molecular weights, the ponderal proportion values were of 26.9% for HA, of 5.1% for sulfo-DBCO-PEG4, and of 68.0% for PNIPAM-N3. HA, hyaluronic acid; NMR, nuclear magnetic resonance; PNIPAM, poly(N-isopropylacrylamide).

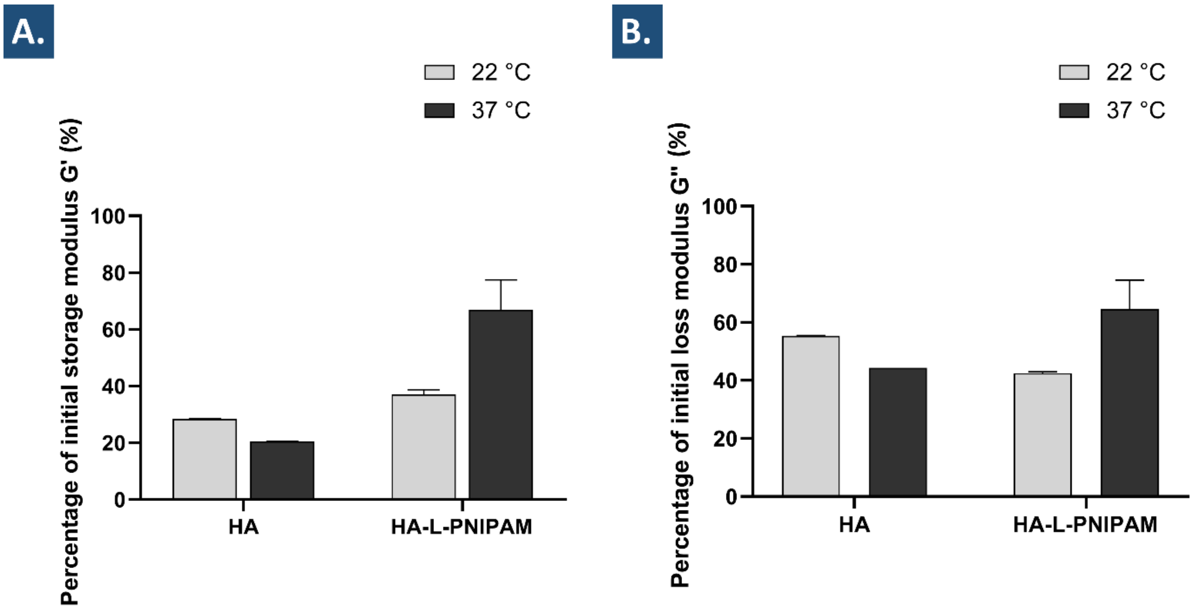

Figure S3. Effects of autoclave sterilization on hydrogels containing HA and HA-L-PNIPAM at the respective concentrations used for the in vitro functional characterization experiments. (**A**) Post-sterilization residual storage moduli G′ and (**B**) post-sterilization residual loss moduli G′′ expressed in percentage of the corresponding values before autoclaving, respectively. HA, hyaluronic acid; PNIPAM, poly(N-isopropylacrylamide).

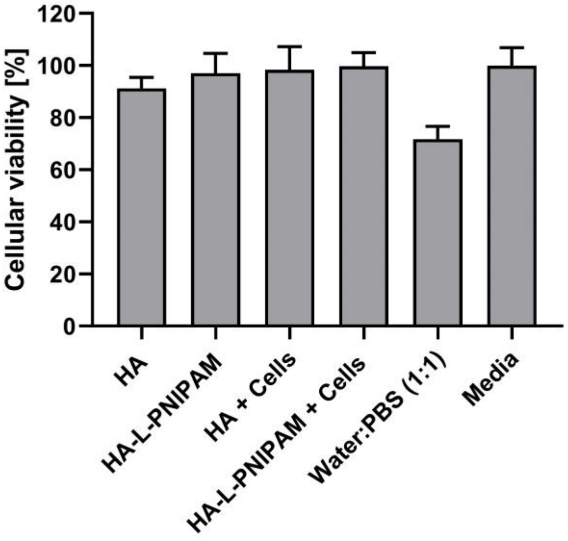

Figure S4. In vitro WST-1 cytotoxicity evaluation of the various combination products with 5 days of sample incubation with human fibroblast-like synoviocytes. HA, hyaluronic acid; PBS, phosphate-buffered saline; PNIPAM, poly(N-isopropylacrylamide).

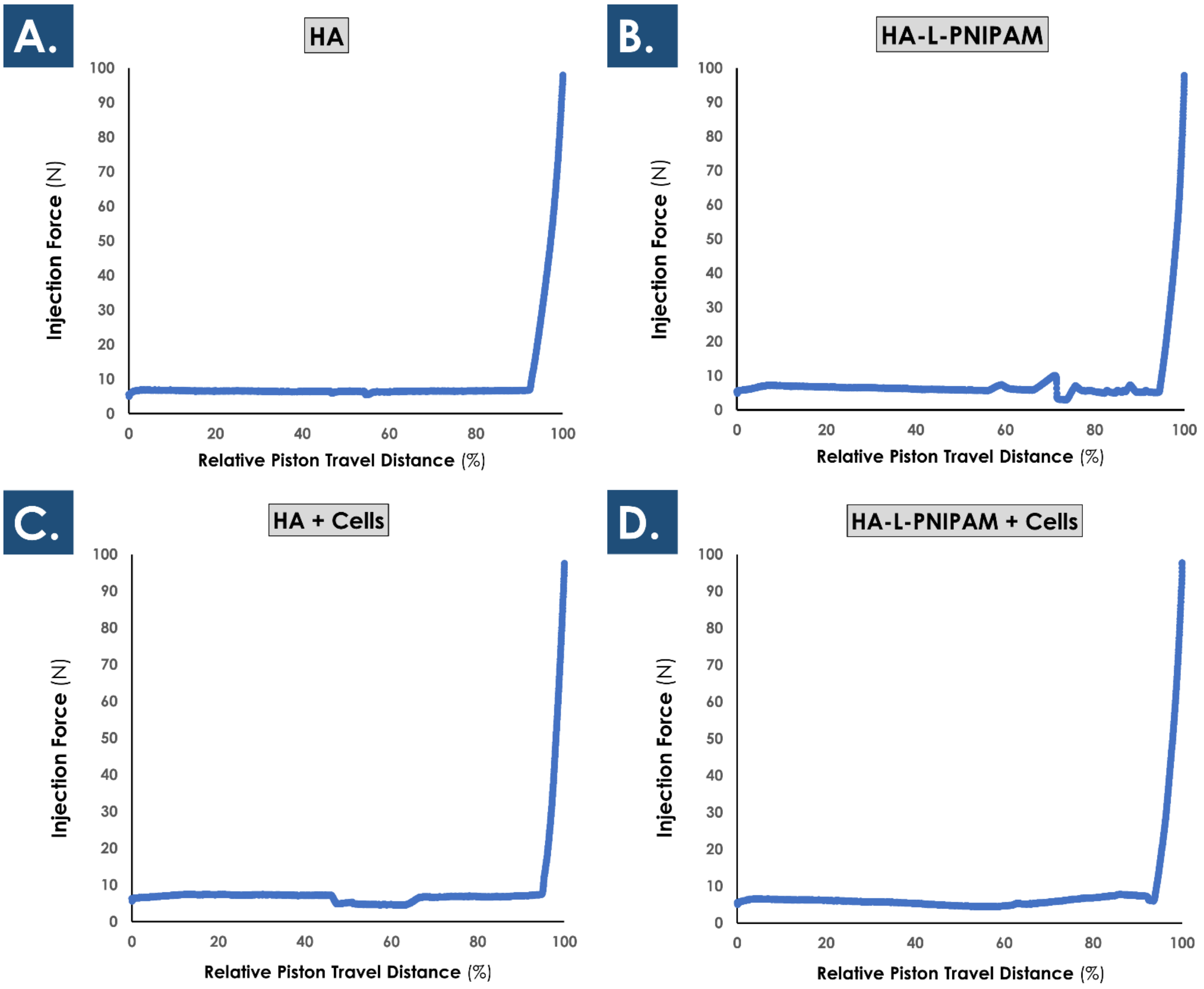

Figure S5. Force injection profiles of both hydrogels with and without FE002 cells, respectively, extruded through 23G gauge needles. (**A**) Force injection profile of the HA-based hydrogel, with a plateau force of 6.44 ± 0.17 N. (**B**) Force injection profile of the HA-L-PNIPAM-based hydrogel, with a plateau force of 6.18 ± 1.02 N. (**C**) Force injection profile of the HA-based hydrogel with FE002 cells, with a plateau force of 6.39 ± 1.08 N. (**D**) Force injection profile of the HA-L-PNIPAM-based hydrogel with FE002 cells, with a plateau force of 5.42 ± 0.64 N. Plateau force values were determined as average forces measured between 20% and 80% of the relative piston travel distance. HA, hyaluronic acid; PNIPAM, poly(N-isopropylacrylamide).

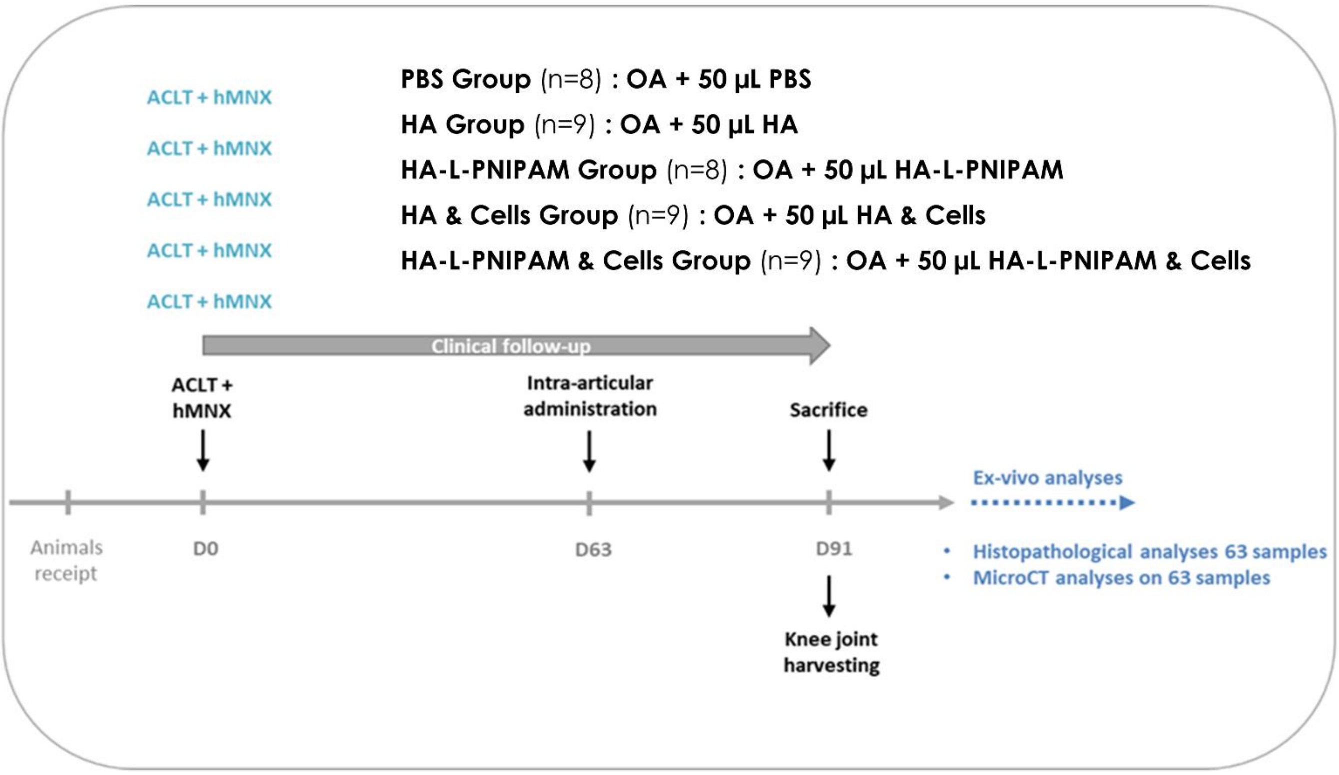

Figure S6. Schematic overview of the preclinical animal study on the applicability and efficacy of the various considered combination products in an adapted model of rat post-ACLT-hMnx knee OA. ACLT, anterior cruciate ligament transection; HA, hyaluronic acid; hMnx, hemi-meniscectomy; OA, osteoarthritis; PBS, phosphate-buffered saline; PNIPAM, poly(N-isopropylacrylamide).

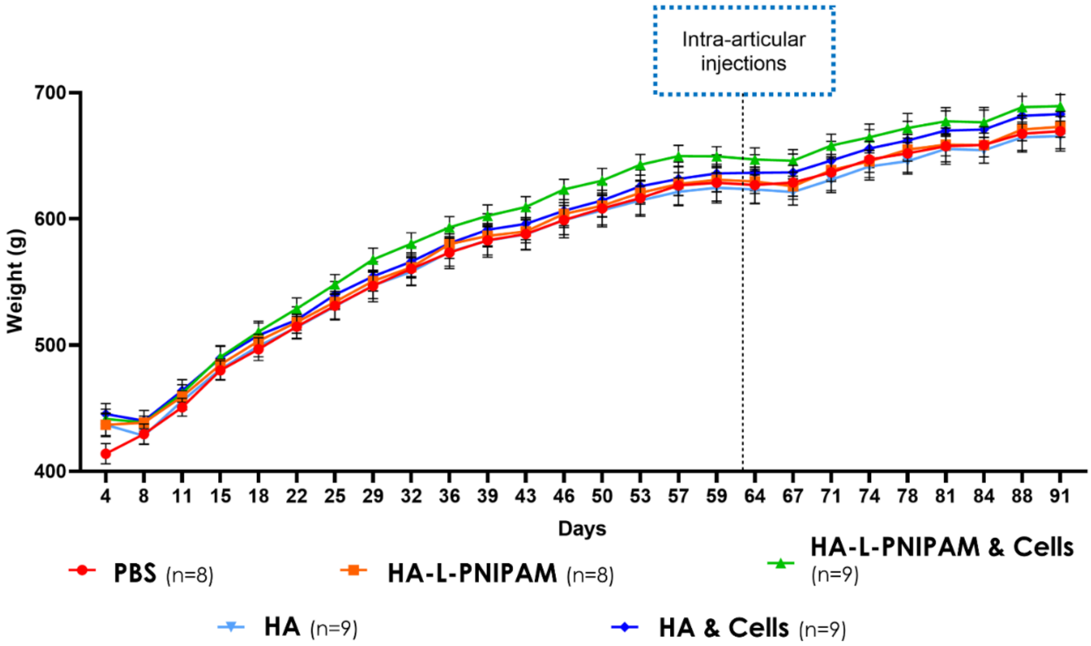

Figure S7. Rat mean body weight curves along the animal study, starting at the time of joint surgical lesion creation (i.e., day 0). Animal weight was recorded twice per week over the length of the in vivo phase. Group 1 corresponded to the PBS condition (i.e., red line), group 2 corresponded to the HA condition (i.e., light blue line), group 3 corresponded to the HA-L-PNIPAM condition (i.e., orange line), group 4 corresponded to the HA + Cells condition (i.e., dark blue line), and group 5 corresponded to the HA-L-PNIPAM + Cells condition (i.e., green line). The results are presented as mean values with standard deviations as error bars for each group. The dotted line represents the day of the intra-articular injections (D63). HA, hyaluronic acid; OA, osteoarthritis; PBS, phosphate-buffered saline; PNIPAM, poly(N-isopropylacrylamide).

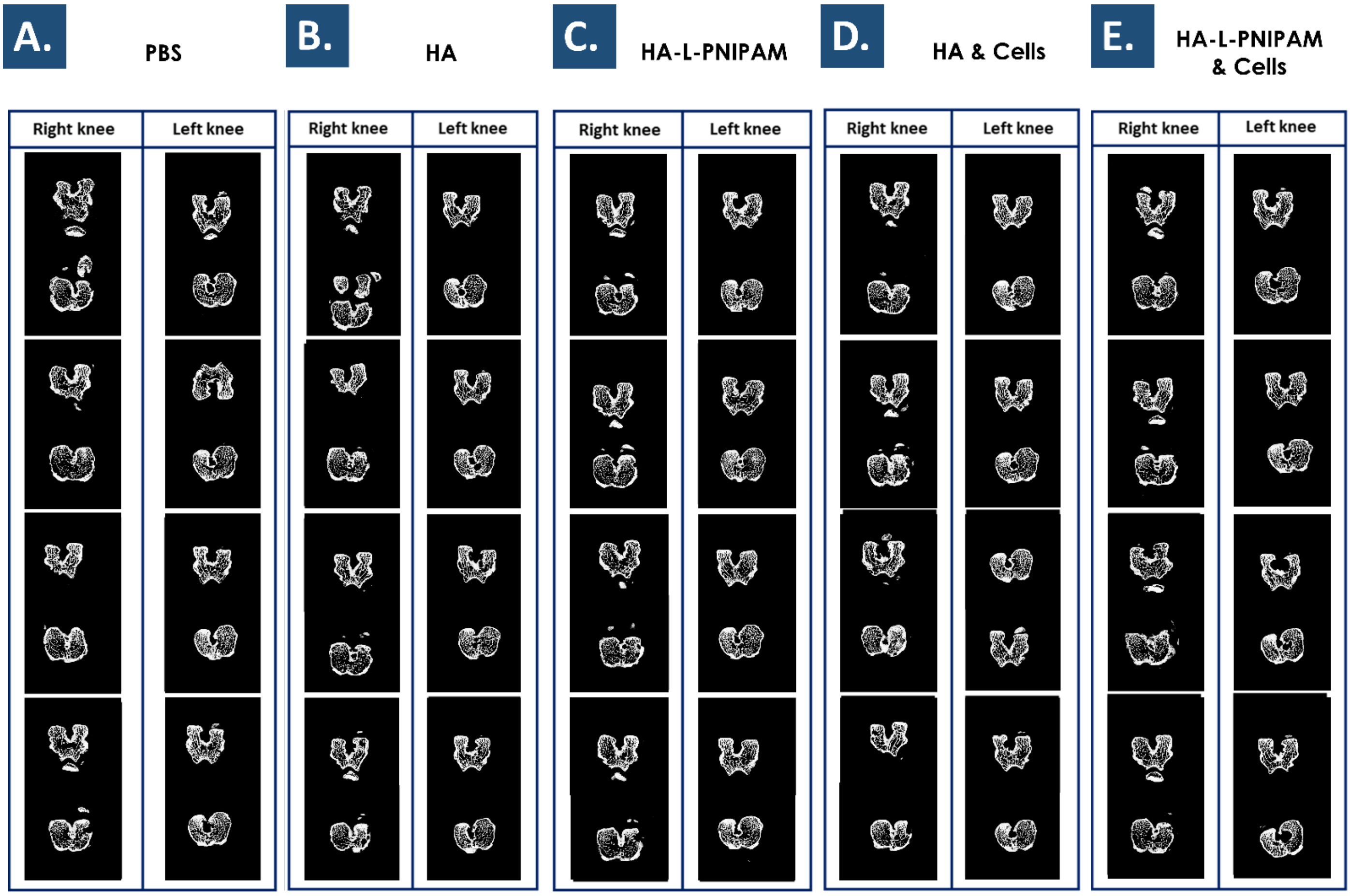

Figure S8. Representative images of transversal sections of femurs and tibiae from the harvested right and left rat knees, which were used for scoring of the presence of osteophytes and bone cysts. The presence of osteophytes and areas of bone cysts were scored on each transversal image of the femur (i.e., top image) and the tibia (i.e., bottom image) for each sample. Illustrative data are presented for the PBS condition (**A**), the HA condition (**B**), the HA-L-PNIPAM condition (**C**), the HA + Cells condition (**D**), and the HA-L-PNIPAM + Cells condition (**E**). Both the operated right knee and the contra-lateral left knee are represented for each group. HA, hyaluronic acid; OA, osteoarthritis; PBS, phosphate-buffered saline; PNIPAM, poly(N-isopropylacrylamide).

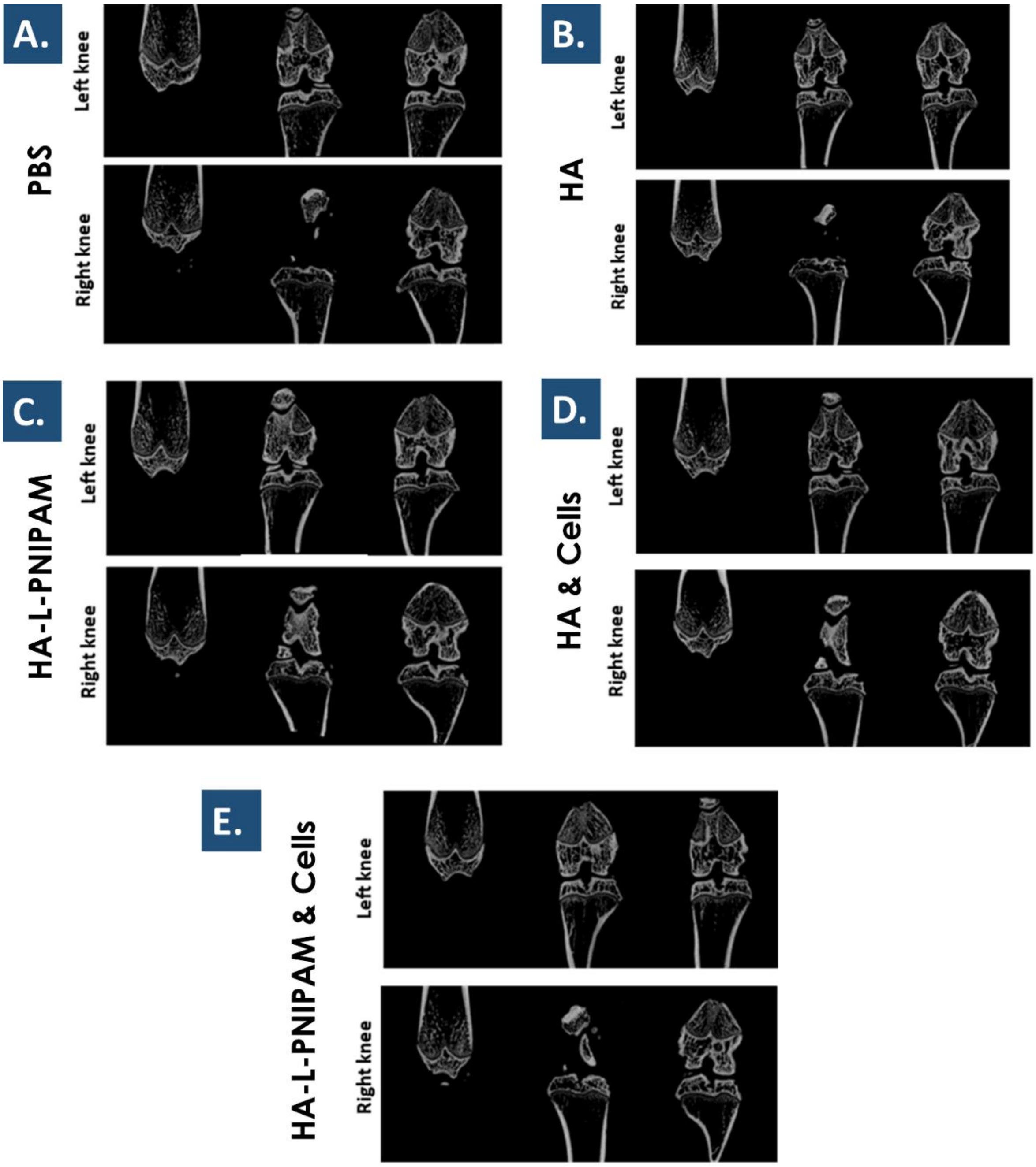

Figure S9. Representative images of the coronal sections of femurs and tibiae, which were used for the scoring of bone sclerosis and bone cysts. For each sample, one image was centered on the femur (i.e., left of the panel), one was centered on the tibia (middle of the panel), and one image comprising both of the aligned structures (i.e., right of the panel) was prepared. Illustrative data are presented for the PBS condition (**A**), the HA condition (**B**), the HA-L-PNIPAM condition (**C**), the HA + Cells condition (**D**), and the HA-L-PNIPAM + Cells condition (**E**). HA, hyaluronic acid; OA, osteoarthritis; PBS, phosphate-buffered saline; PNIPAM, poly(N-isopropylacrylamide).

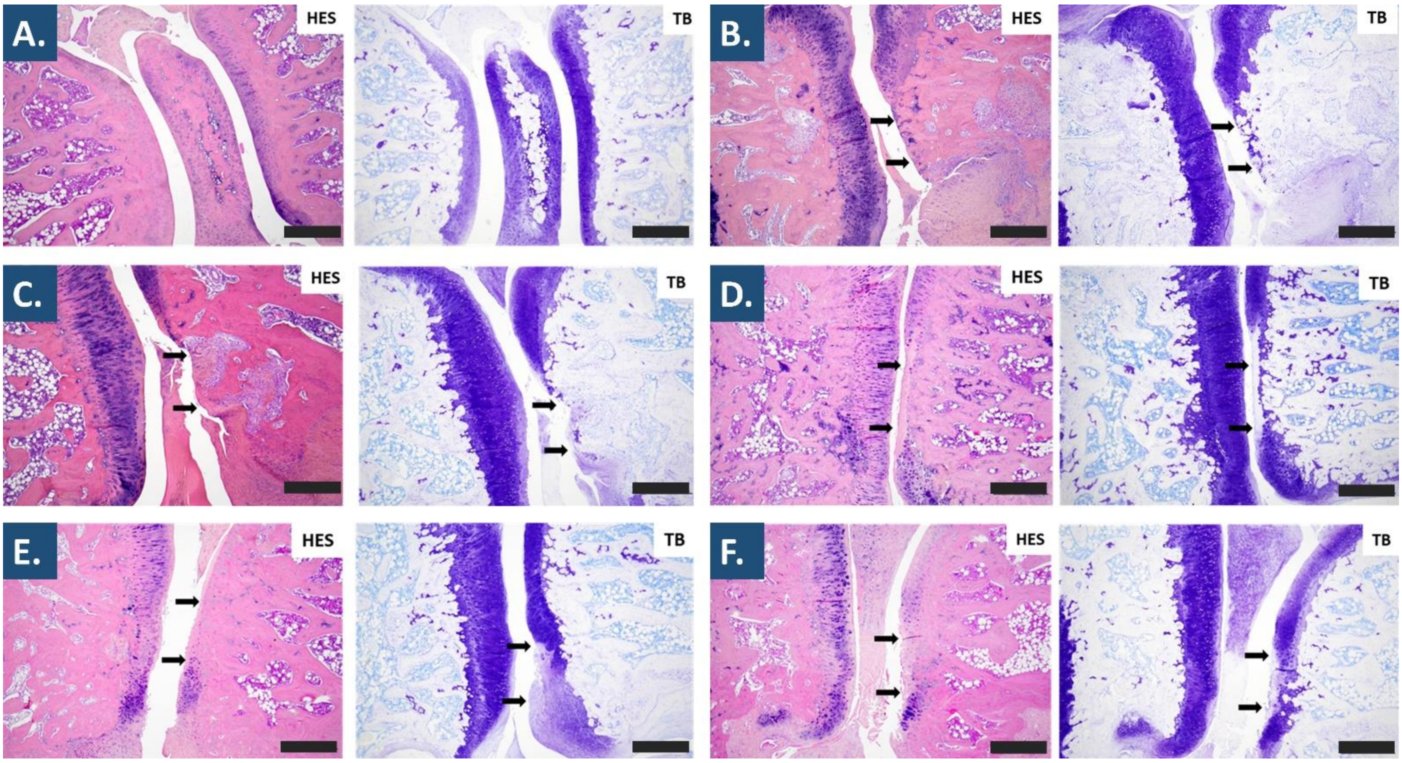

Figure S10. Histological representative images of a non-operated knee from the PBS control group (**A**), of an operated knee from the PBS control group (**B**), of an operated knee from the HA group (**C**), of an operated knee from the HA-L-PNIPAM group (**D**), of an operated knee from the HA + Cells group (**E**), and of an operated knee from the HA-L-PNIPAM + Cells group (**F**). The left portions of the panels correspond to Hematoxylin/Eosin/Saffron (HES) staining and the right portions of the panels correspond to Toluidine Blue (TB) staining. Scale bars = 200 μm. HA, hyaluronic acid; PBS, phosphate-buffered saline; PNIPAM, poly(N-isopropylacrylamide).

Supplementary Tables

Table S1. Grading results of the produced lyophilized FE002 chondroprogenitors. Material assessments and semi-quantitative gradings were performed by two experienced operators using the nomenclature presented hereafter. (–) = unsatisfactory; (+) = sub-optimal; (++) = satisfactory; (+++) = optimal. All quantitative measurements were performed in triplicate and gradings were performed on at least three separate vials from a production batch.

Parameters	Targets/Acceptance Criteria	Grading Results
Presence of cake	Presence of a solid cake	+++
Batch uniformity	Uniform lyophilizate batch	+++
Cake color	White cake color	+++
Cake structure	Uniform cake structure	+++
Cake density	Dense cake	+++
Cake finish	Shiny or sheen cake finish	++
Cake friability	Non-friable cake	+++
Cake topography	Consistent cake topography	++
Cake shrinkage	Minimal cake shrinkage	++
Cake collapse/Meltback	No cake collapse or meltback	+++
Residual material on vial	Minimal residual material presence on the vial	++
Adventitious material presence	Absence of observable adventitious material presence	+++
Cake resuspension time	Total resuspension of the cake < 90 seconds	+++
Residual moisture level	Residual moisture level < 8% and > 1% water	+++
Observable microscopic particle presence	Observable microscopic particle presence in reconstituted samples in contrast phase microscopy (400X optical enlargement)	+++
pH value	pH value of reconstituted samples of 7.0 ± 1.25	+++
Osmolality value	Osmolality value of reconstituted samples of 300 ± 30 mOsmol/kg	+++

Table S2. Scoring table for the presence of osteophytes on the femurs and tibiae from CT-scan data.

Score	Criteria
0	None
1	Defined but focal
2	Extended

Table S3. Scoring table for the presence of bone cysts on the femurs and tibiae from CT-scan data.

Score	Criteria
0	None
1	Occupying around 1/4 of the area
2	Occupying around 1/3 of the area
3	Occupying around 1/2 of the area
4	Occupying more than 1/2 of the area

Table S4. Scoring table for the presence of bone sclerosis on the femurs and tibiae from CT-scan data.

Score	Criteria
0	None
1	On less than 1/2 of the subchondral or cortical epiphyseal bone
2	On 1/2 of the subchondral or cortical epiphyseal bone
3	On more than 1/2 of the subchondral or cortical epiphyseal bone

Table S5. Scoring table for articular cartilage structure and proteoglycan content evaluation from histological data.

Parameter	Grade	Description
Articular Cartilage Structure	0	Normal, smooth, uninterrupted surface
	1	Mild surface irregularities (i.e., undulations)
	2	Irregular surface, 1–3 superficial clefts (i.e., fissures)
	3	> 3 fissures and/or loss of cartilage in the superficial zone
	4	1–3 fissures extending into the middle zone
	5	> 3 fissures and/or loss of cartilage extending into the middle zone
	6	1–3 fissures extending into the deep zone
	7	> 3 fissures extending into the deep zone and/or loss of cartilage to the deep zone
	8	Fissures or loss of cartilage extending to the zone of calcified cartilage
Proteoglycan Content	0	Uniform throughout articular cartilage
	1	Decreased in the superficial zone only and for < half of the length of the condyle or plateau
	2	Decreased in the superficial zone for half of the length or greater of the condyle or plateau
	3	Decreased in the superficial and middle zones for < half of the length of the condyle or plateau
	4	Decreased in the superficial and middle zones for half of the length or greater of the condyle or plateau
	5	Decreased in all 3 zones for < half the length of the condyle or plateau
	6	Decreased in all 3 zones for half the length or greater of the condyle or plateau
